# Benchmarking large-scale single-cell RNA-seq analysis

**DOI:** 10.1101/2025.10.28.681564

**Published:** 2025-10-29

**Authors:** Ilaria Billato, Herve Pages, Vince Carey, Levi Waldron, Gabriele Sales, Chiara Romualdi, Davide Risso

**Affiliations:** 1 Department of Biology, University of Padova, via Ugo Bassi 47, Padova, 35132, Italy.; 2 Fred Hutchinson Cancer Research Center, Seattle, WA, USA.; 3 Channing Division of Network Medicine, Brigham and Women’s Hospital and Harvard Medical School, Boston, MA, USA.; 4 Department of Epidemiology and Biostatistics and Institute for Implementation Science in Public Health, CUNY Graduate School of Public Health and Public Health Policy, 55 W 125th St, New York, 10027, NY, USA.; 5 Department of Statistical Sciences, University of Padova, via Cesare Battisti 241, Padova, 35131, Italy.

**Keywords:** Single-cell RNA-seq, benchmark, scalability

## Abstract

The increasing size of single-cell RNA sequencing (scRNA-seq) datasets poses major computational challenges. This work benchmarks the scalability, efficiency, and accuracy of five widely used analysis frameworks (Seurat, OSCA, scrapper, Scanpy, and rapids_singlecell), focusing on the impact of algorithmic and infrastructural choices on performance. We performed a systematic comparison of these workflows using representative datasets, including a 1.3 million mouse brain cell dataset for scalability and three smaller datasets (BE1, scMixology, and cord blood CITE-seq) with ground truth labels to assess clustering accuracy. Principal Component Analysis (PCA) was used as a paradigmatic step to evaluate the computational performance of six SVD algorithms (exact, ARPACK, IRLBA, randomized, Jacobi, and incremental PCA) across multiple data representations (dense, sparse, HDF5) and hardware configurations (CPU vs GPU). All methods showed high concordance in PCA results, with negligible loss of accuracy in truncated approaches. GPU-based computation using rapids_singlecell provided a 15× speed-up over the best CPU methods, with moderate memory usage. On CPU, ARPACK and IRLBA were the most efficient for sparse matrices, while randomized SVD performed best for HDF5-backed data. Among full pipelines, rapids_singlecell was the fastest, whereas OSCA and scrapper achieved the highest clustering accuracy (ARI up to 0.97) in datasets with known cell identities. Performance differences were largely driven by the choice of highly variable genes (HVGs) and PCA implementation. The study highlights that scalability in scRNA-seq analysis depends critically on both algorithmic and infrastructural factors. GPU acceleration and optimized BLAS/LAPACK configurations markedly enhance performance, while Bioconductor-based pipelines remain robust in accuracy. The provided benchmarks offer practical guidelines for efficient and reliable analysis of large-scale single-cell datasets.

## 1 Introduction

As single-cell RNA sequencing (scRNA-seq) reaches its teenage years [1], we are witnessing a rapid increase in the size and complexity of experiments and datasets [2]. In fact, whereas early scRNA-seq comprised hundreds to few thousands cells, typically in a single condition and often without biological replicates [3], contemporary experiments include cells from multiple individuals measured across conditions (e.g., treatments, genotypes, health states). Often, researchers target 5 – 10,000 cells per replicate and the final dataset easily comprises hundreds of thousands of cells (e.g., [4, 5]). Furthermore, single-cell atlases have become mature and thanks to programmatic access, it is now straightforward to download and locally analyse datasets made of millions of cells from multiple organs across different labs [6, 7].

This increasing complexity poses significant computational challenges throughout the analysis pipeline, from data preprocessing to downstream interpretation. These challenges are exacerbated by the exploratory nature of many scRNA-seq analyses, which need to process large datasets in an interactive way, e.g., trying different analysis paths and exploring the downstream results, often using desktops or laptops rather than high-performance computing (HPC), requiring frugal and efficient workflows.

A typical scRNA-seq analysis workflow starts with gene expression quantitation, a process that includes assigning reads to barcodes, aligning reads to the appropriate genome or transcriptome, and quantifying gene expression by counting Unique Molecular Identifiers (UMIs) assigned to each gene. This process is typically performed in HPC clusters and is usually carried-out with stand-alone software or standardized pipelines. This step is typically not part of the interactive process described above; hence for the sake of this article, we will consider the output of this step as the starting point of the analysis. Interested readers can refer to the literature for a benchmark of the different preprocessing pipelines (e.g., [8]).

**Fig. 1:**
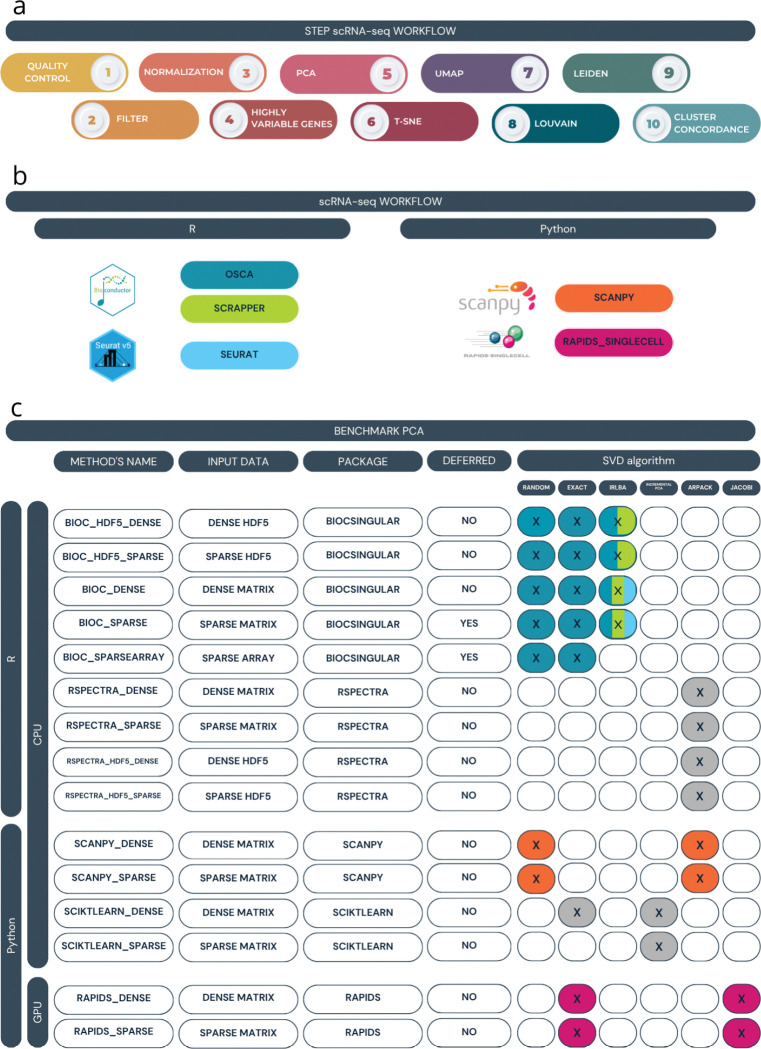
Overview of PCA benchmarking and single-cell workflow comparison. (a) Schematic of the typical steps in single-cell data processing workflows, including quality control, filtering, normalization, highly variable genes selection, dimensionality reduction (PCA, UMAP, t-SNE), clustering (Louvain, Leiden), and cluster concordance assessment. (b) List of R and Python-based single-cell RNA-seq analysis frameworks used for comparing full workflows: OSCA, Scrapper, and Seurat (R); Scanpy and rapids_singlecell (Python).(c) Summary of PCA implementations evaluated in the benchmark, categorized by programming language (R or Python), computation type (CPU or GPU), input data format (dense matrix, sparse matrix, or HDF5), library name, and support for deferred computation. The table also indicates the supported SVD algorithms for each method (random, exact, IRLBA, Incremental PCA, ARPACK, Jacobi). The colors represent whether the methods are implemented in the frameworks of panel (c) or not (grey).

After preprocessing, the analysis workflow comprises the following steps (Fig. 1a): (i) quality control; (ii) gene and cell filtering; (iii) normalization; (iv) identification of highly variable genes; (v) dimensionality reduction, generally employed using principal component analysis (PCA) or similar methods; (vi) data visualization, using methods such as t-SNE and UMAP, which are commonly applied to data reduced through PCA; (vii) clustering for the identification of groups of cells with similar transcriptional profiles; and (viii) cell type annotation, the process by which cells or clusters are labeled using either external reference datasets or the expression of known marker genes [9].

Each of these steps requires its own processing time and memory usage and, in the last few years, several approaches have been proposed in different programming languages (mostly R and Python).

While there have been attempts in the literature to benchmark individual analysis steps, such as normalization, dimensionality reduction, clustering, trajectory inference, cell-type annotation, data integration [10–15], the comparison of entire analysis workflows has received less attention. Rich et al. [16] explores the impact of parameter choice and their default values in Seurat and Scanpy, but does not thoroughly examine their scalability. On the other hand, Tian et al. [17], while benchmarking all the different steps of a typical analysis, does not consider the workflow typically recommended by the most popular frameworks. Moreover, the dataset used for benchmarking is relatively small and insufficient to tackle scalability issues.

Here, we focus on the three most popular analysis frameworks, namely, Bioconductor [9], scverse/Scanpy [18], and Seurat [19] (Fig. 1b). For Bioconductor, we consider both the standard Orchestrating Single-Cell Analysis (OSCA) workflow and scrapper, a more recent efficient implementation that offloads most computations to C++ (see Methods). To compare these workflows, we rely on their respective documentation, purposely choosing the “quick start” or “basic” tutorials, which comprise the steps that are always present in one’s analysis; more advanced methods, such as multi-sample integration, batch-effect reduction, reference-based annotations, are not included in our benchmark.

While traditional approaches rely on CPU architectures, although often allowing for multithreading and parallel computing, recent efforts have explored the use of GPUs to enhance speed and scalability [20]. Hence, we include in our benchmark the rapids_singlecell framework [21], an open-source library that implements a standard single-cell analysis workflow leveraging NVIDIA GPUs (Fig. 1b). In this context, benchmarking tools and workflows is essential for evaluating the performance of different methods in different experimental settings (such as the number of cells, sequencing depth, or biological complexity) not just in terms of efficiency, but to ensure that scalability does not come at the price of reduced accuracy.

The goal of this article is three-fold: first, we aim to benchmark the five above-mentioned scRNA-seq workflows, namely Seurat, OSCA, scrapper, Scanpy, and rapids_singlecell; second, by focusing on PCA as an exemplar analysis step, we explore in greater details how different algorithms and input choices can affect the efficiency and scalability of methods; last, we explore efficient workflows, in CPU and GPU, establishing guidelines and recommendations for the user in need of efficient analysis of large data.

## 2 Results

### 2.1 Benchmarking PCA

While quality control, normalization, and clustering are all essential step in single-cell data analysis, we chose to focus on PCA as an exemplar step to thoroughly benchmark. In fact, PCA represents a key focal point of the entire pipeline as it is a non-trivial, often computationally heavy step, necessary for the subsequent stages of the analysis. Moreover, several algorithms exist to compute PCA and the choice of the algorithm can have profound impact on its computational load [15].

First, we give a brief introduction to PCA, reviewing standard methods for computing the principal components of a data matrix, i.e., the Singular Value Decomposition (SVD), as well as some of the most popular approximations, based on truncated SVD.

Briefly, PCA is a multivariate technique that consists of defining a new coordinate system defined by a set of orthogonal vectors, which correspond to the direction of maximal variability in the data. The principal components (PCs) are hence a set of linear combination of the original variables (in our case the genes) that explains the most variation in the data.

PCA is typically used as a dimensionality reduction technique, in which the first few components are retained and the data are projected onto their subspace. PCA ensures that this is the “best” linear subspace, in the sense that it preserves the most variation in the data.

In mathematical terms, the principal components are the eigenvectors of the covariance matrix and can be computed by its eigendecomposition. Alternatively, the PCs can be computed directly by the SVD of the original expression matrix. We refer the reader to [22] and [23] for a more thorough introduction and the mathematical details.

SVD is a standard matrix factorization technique, and many algorithms exist for its computation (see e.g., [24]). However, when only the first few PCs are needed, approximated methods, collectively known as truncated or partial SVD, are computationally advantageous (see Methods for details).

When choosing the appropriate SVD algorithm, the input format may play an important role. In fact, it is likely that an algorithm that very efficiently computes the first k principal components when the data are stored in RAM memory, becomes highly inefficient when the data are stored out of RAM memory, as in the case of delayed operations on HDF5-backed datasets [25–27]. Similar considerations may be made for dense versus sparse matrix representations.

In our benchmark, we compared the computational time and RAM memory usage of six SVD algorithms, implemented in R and Python, applied to three different input formats for a total of 28 combinations (Fig. 1c and Supplementary Table S2). The process was applied to four subsets of the 1.3M cell dataset of increasing size and repeated ten times for both time and memory assessments, for a total of 2,240 SVD calculations.

In particular, the SVD algorithms considered include ARPACK [28], random [29], exact [30], IRLBA [31], Jacobi [32], and incrementalPCA [33], with technical details provided in the Methods section. In R, we used the BiocSingular [34] and Rspectra [35] packages, while in Python, we relied on Scanpy [18], scikit-Learn [36], and RAPIDS [20] (Fig. 1c).

Several input data formats were considered: the data were stored either in an HDF5 [25] file or in RAM memory, and in each case they were stored as dense or sparse matrices. We compared these input formats with the *SparseArray* representation [37] (Fig. 1c). This exploration allowed us to discern how the choice of input data influenced the results. For sparse matrices, whenever possible, we employed the “deferred” option, which postpones centering and scaling until after matrix multiplication to leverage the sparsity of the matrix for as long as possible.

To evaluate the scalability, we considered three datasets (random subsamples of the 1.3M cell dataset [38]) with the same genes but with an increasing number of cells: 100k, 500k, 1M (see Methods for details). To ensure the robustness of the results, each method was executed 10 times on the same machine, using a single core, mitigating potential biases arising from parallel computation.

Indeed, certain algorithms are optimized for parallel processing, while others are not (Supplementary Fig. S1b). In some cases, increasing the number of cores does not enhance computational performance. For example, with the IRLBA algorithm, parallel processing results in a substantial increase in computation time, rising from 0.39 minutes with a single core to 22.56 minutes with two cores (Supplementary Fig.S1a).

Hereafter, the term “method”, which indicates the combination of algorithm and input type, is used as the comparison unit. The names of the methods follow this structure: package name + input data type + deferred (optional) + SVD algorithm.

#### 2.1.1 Accuracy of truncated SVD

Before analyzing the performance in terms of computational time and memory usage, we evaluate the degree of agreement among the different methods to ensure that the faster algorithms return a good approximation of the true principal components. We measured the consistency across methods by computing the correlation between principal components (PCs). Because PCA is rotation-invariant and the singular vectors are defined only up to sign changes, we report the absolute value of the correlation when comparing components, using the exact SVD algorithm as the ground truth.

In single-cell analysis, the number of selected PCs can vary substantially depending on the dataset and analytical requirements. However, a maximum of 50 PCs are often assumed to capture sufficient variability. Hence, we consider here only the first 50 PCs.

**Fig. 2:**
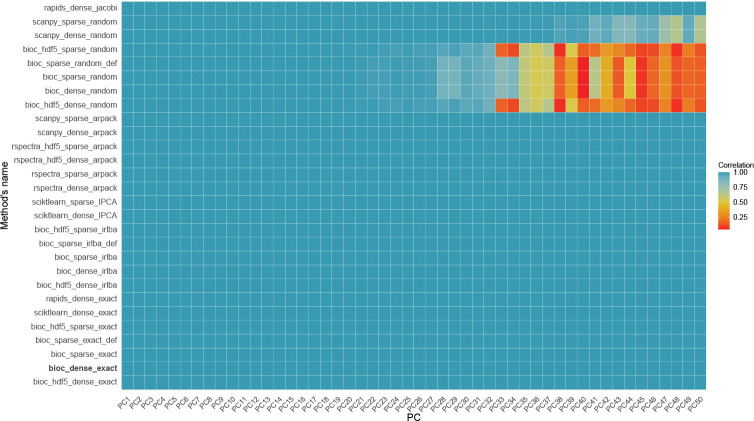
Correlation heatmap between principal components (PC) Correlation heatmap between principal components (PCs) obtained using different PCA algorithms and the Exact Algorithm as reference on 1.3M cell dataset. Each row represents a different combination of PCA algorithm and input matrix, and each column a principal component (PC1–PC50). The color indicates the absolute Pearson correlation between the PC obtained from each method and the corresponding PC from the exact reference.

Figure 2 shows the correlation between exact SVD (bioc_dense_exact) and all the other methods in the 1.3M cell dataset. Strikingly, the correlation between exact SVD and most other methods is exactly equal to 1 for all considered PCs. The only exception is random SVD, which, especially in its R implementation, shows a significant decline in correlation values (up to values close to 0) starting from the 35th PC. This result implies that if one expect important information to be captured by the later PCs, random SVD is a poor algorithmic choice. However, it is important to note that typically the variance explained by the 35th and later PCs is very low. For instance, in the 1.3M cell dataset, the PCs from 35 to 50 in bioc_dense_random explain only 0.2% of the total variability (Supplementary Tables S11–S15). Hence, this result is unlikely to have a large practical importance for real data analysis. Supplementary Figure S2 shows the same analysis on random subsets of 100k, 500k, 1M cells.

#### 2.1.2 Computational time and memory usage

**Fig. 3:**
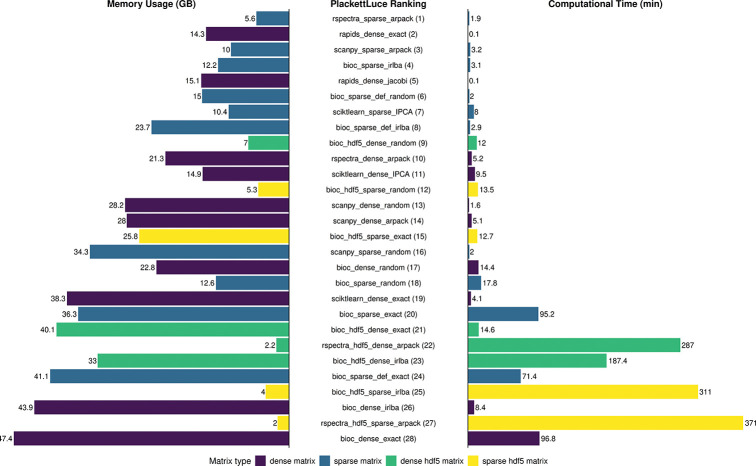
Comparison of PCA method performance across input matrix types for 1.3M cell dataset. Barplots show (left) peak memory usage (GB), (center) Plackett–Luce ranking (lower is better), and (right) computational time (minutes) for the 1.3M cell dataset. Each bar represents a specific combination of SVD algorithm and matrix format. Colors indicate the type of input matrix: dense matrix (purple), sparse matrix (blue), dense HDF5 matrix (green), and sparse HDF5 matrix (yellow).

Figure 3 reports the time (left side) and memory consumption (right side) for each combination of SVD algorithm implementation, computational platform, and input type for the 1.3M cell dataset (see Supplementary Tables S6 and S10 for the exact numerical results).

The GPU-aware RAPIDS library is the fastest approach, independent of the SVD algorithm, being at least one order of magnitude faster than CPU methods. Its memory footprint is moderate, even though it works only on dense matrices, providing an excellent choice for those that have a GPU available. In fact, it only takes 7.5 seconds to compute the exact SVD of a matrix with 1.3 million cells (Fig. 3, Supplementary Table S6).

The performance of exact SVD using CPU is surprisingly different in stock installations of Python and R. Indeed, Python’s scikit-learn implementation of exact SVD is 20 times faster than R’s base *svd* function (Fig. 3, Supplementary Table S6), despite both functions calling the same underlying LAPACK routine in FORTRAN (*gesdd*). This surprising result can be explained by the different LAPACK configuration between the two systems: R’s default BLAS/LAPACK on many systems is a reference implementation, which is not optimized for performance. Python, especially in environments like Conda [39], usually links to highly optimized libraries like OpenBLAS [40] or MKL (Intel Math Kernel Library). These optimized libraries use hardware-specific vectorization and threading to speed up linear algebra. In fact, running R’s exact SVD with optimized OpenBlas results in a 15X speed up, leading to computing times similar to those of Python (Supplementary Table S16).

When the input matrix is large and one does not need the full singular values/vectors, truncated SVD algorithms are advantageous compared to computing the exact SVD, both in terms of speed and memory. The fastest approaches are random SVD as implemented in Scanpy, both applied to dense and sparse matrices (1.6 and 2 minutes, respectively, Fig. 3, Supplementary Table S6) and the ARPACK algorithm applied to sparse matrices, especially as implemented in the RSpectra package (1.9 minutes, Fig. 3, Supplementary Table S6). Notably, using a sparse matrix representation as input saves RAM memory, using e.g., 5.6GB for RSpectra sparse, compared to 21.3GB for RSpectra dense. Similar considerations can be made for ARPACK in Scanpy and random SVD in BiocSingular, but surprisingly random SVD in Scanpy uses more RAM memory for sparse matrices than for dense matrices, perhaps due to the fact that centering the matrix prior to SVD removes sparsity. Note that BiocSingular applies centering in a deferred way to preserve sparsity for the SVD calculations.

IRLBA is a fast alternative for truncated SVD for in-memory matrices, especially when represented in a sparse format (3.1 minutes, Fig. 3, Supplementary Table S6). However, IRLBA and ARPACK are both very slow when applied to out-of-memory data, exceeding 1.5 hours when applied to HDF5 matrices. This is likely due to the multiple passes on the data needed by these algorithms, which make them suffer from the I/O bottleneck. On the other hand, exact and random SVD perform better for HDF5 than for in-memory inputs, making it the best choice for out-of-memory data: e.g., random SVD applied to dense HDF5 data takes 12 minutes.

We next used the Plackett-Luce model [41, 42] to establish a single, final method ranking, encompassing computational time, memory usage, and the average correlation among the top 50 principal components (Fig. 3). The ranking clearly highlights four methods that outperform all the other: ARPACK sparse (both implemented in RSpectra and Scanpy), IRLBA sparse, and Rapids dense (both exact and Jacobi).

**Fig. 4:**
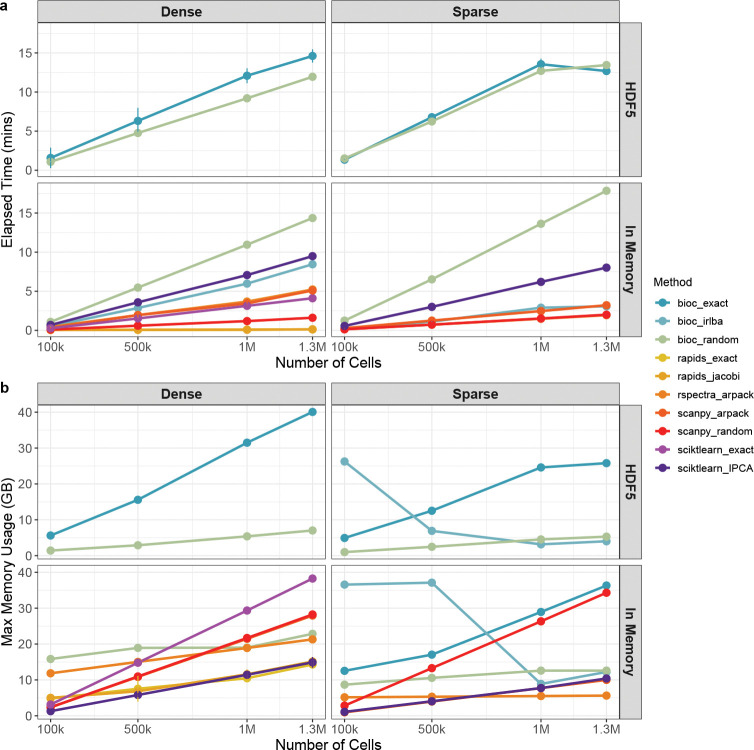
Scalability Assessment of PCA Methods by Input Dimensions, Runtime, and Memory Consumption (a) Elapsed time (in minutes) required to perform principal component analysis (PCA) across a range of dataset sizes (100k, 500k, 1M, 1.3M cells), using different combinations of methods, matrix formats (dense or sparse), and storage types (in-memory or HDF5). Only methods with a maximum average execution time below 75 minutes are shown. (b) Maximum memory usage (in GB) required to perform principal component analysis (PCA) across a range of dataset sizes (100k, 500k, 1M, 1.3M cells), using different combinations of methods, matrix formats (dense or sparse), and storage types (in-memory or HDF5). Only methods whose maximum memory usage did not exceed 42 GB are shown.

Finally, we assessed the scalability of each algorithm by taking random subsets of the 1.3M cell dataset and evaluate the time and memory consumption of each method across dataset sizes (Fig. 4, Supplementary Fig. S3). In terms of time, all methods scale linearly with the number of cells. Strikingly, with dense input matrices Python methods are more scalable than R methods. This is largely due to the availability of optimized BLAS libraries, which are not always installed by default across different operating systems. Linking R to an optimized BLAS/LAPACK almost entirely accounts for this difference (see Supplementary Table S16). On the other hand, R is very efficient at handling sparse matrices, thanks in part to the BiocSingular ability to defer centering and scaling (Fig. 4a, Supplementary Fig. S3, Supplementary Tables S3–S5). The IRLBA algorithm is by far the slowest algorithm when the input is out of memory, due to the multiple read accesses (Supplementary Fig. S3; see Methods).

In terms of RAM memory, as expected, sparse matrices lead on average to lower usage than dense matrices (Fig. 4b, Supplementary Fig. S3, Supplementary Tables S7–S10). Interestingly, random SVD and ARPACK show better RAM scalability than other methods, as evident from the lower slope of the lines (Fig. 4b); this confirms that for larger data sets ARPACK is a good choice. A surprising pattern is observed for IRLBA on sparse matrices (both in and out of memory): RAM usage is much higher for low-dimensional matrices than for much higher dimensions. The IRLBA algorithm involves multiple read access to the input matrix (two per iteration; see Methods) and caching in memory may be employed by the algorithm for smaller matrices to improve computational time.

Taken together, these results show that the choice of the algorithm is critically dependent on the input data and the computing infrastructure. If the data are small enough to fit in the GPU’s VRAM, this is by far the most computationally efficient method; if one cannot use a GPU, but the data fit in the CPU’s RAM, ARPACK, random SVD, and IRLBA are all good choices. If the data are sparse, RSpectra is the fastest and most memory-efficient implementation, while if the data are dense, Scanpy’s random SVD method is the fastest, while incremental PCA is the most memory-efficient. Finally, when the data are out-of-memory, the best approach is random SVD as implemented in BiocSingular, which results in a reasonable time and a low memory footprint.

### 2.2 Single-cell RNA-seq Workflow

We now shift our focus to evaluating single-cell analysis pipelines in their entirety. We assess each pipeline using its standard analysis steps as recommended by the developers.

In addition to computational time and memory usage, we aim at comparing each workflow’s accuracy. To do so, we selected three datasets that contain different degrees of ground truth, which allows us to evaluate the ability of the workflows to capture real biological characteristics of the analyzed data (Supplementary Table S1).

In particular, we tested the pipelines using the BE1 [43] and sc_mixology [17] datasets, composed of mixtures of cell lines: the cell line of origin of each cell is recorded, making these data useful to evaluate the ability of the workflows to recover known cell clusters. In addition, we use a Coord Blood CITEseq dataset [44], which, in addition to scRNA-seq, includes cell surface markers that allow the manual gating of cells for cell typing independent of scRNA-seq; treating these cell types as ground truth, we evaluate the ability of the workflows to recover them using only the scRNA-seq profiles. Specifically, we evaluate the concordance of clustering results, measured by the Adjusted Rand Index (ARI) and the cell-type separation in PCA space (see Methods for details).

While useful for the presence of ground truth, none of the previously described datasets are “large”, comprising only a few thousand cells. Hence, to study the computational efficiency and scalability of the methods, we employ the 1.3M cell dataset from 10x Genomics [38]. Taken together, these datasets allow us to test the workflows on different organisms, data throughput, and sample types (Supplementary Table S1).

**Fig. 5:**
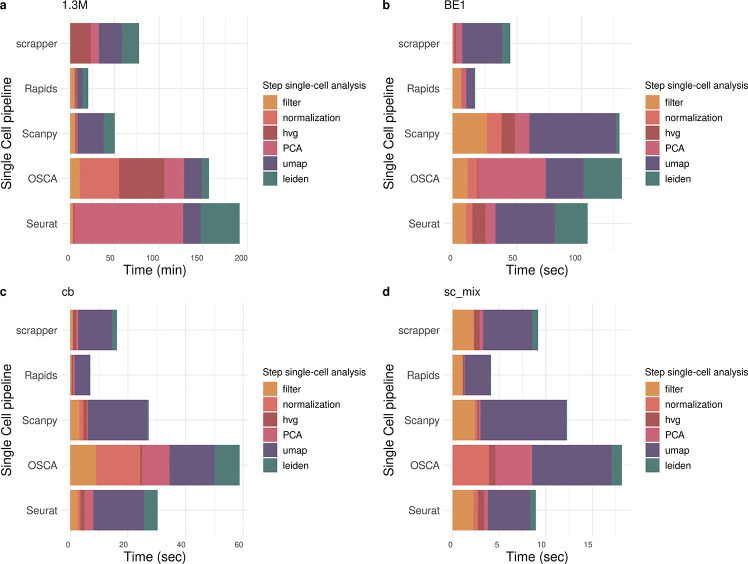
Barplot of elapsed time for each dataset used in the workflow benchmark. (a) Each panel (a–d) displays the computational time required by different single-cell analysis pipelines across various datasets: (a) 1.3M cells, (b) BE1, (c) cb, and (d) sc_mixology. Pipelines include Seurat, OSCA, Scanpy, Rapids, and Scrapper. Bars are colored by processing step: filtering, normalization, selection of highly variable genes (HVG), PCA, UMAP, and Leiden clustering. Time is expressed in minutes for the 1.3M cell dataset and in seconds for the others.

The time in seconds for each step of the analysis is reported in Supplementary Table S17 and in Figure 5 for each dataset. As expected the computational time is proportional to the dimension of the dataset ranging from a few seconds for sc_mixology to more than 2 hours for the 1.3M cell dataset.

As expected, the RAPIDS single-cell pipeline is the fastest across all datasets, highlighting the potential of leveraging GPU acceleration for single-cell analysis, as already reported [20]. On the other hand, Seurat and OSCA exhibit the largest computational times. This is particularly evident in the 1.3M cell dataset, with RAPIDS taking about 20 minutes and OSCA and Seurat taking about two and a half and three hours, respectively (Fig 5a). In this dataset, Seurat’s computational performance is hindered by PCA and Leiden clustering, while OSCA’s computational bottleneck is upstream, normalization and highly variable gene (HVG) selection being the most computationally heavy steps (Fig. 5a).

For smaller datasets, the gaps are less important, given that all workflows are able to finish the analysis in minutes, if not seconds. Nonetheless, the general trend is confirmed (Fig. 5b–d).

We next turned to evaluating the accuracy of the clustering derived from each pipeline: Table 1 shows the ARI between the clustering derived from each workflow and the ground truth cell labels. Notably, all workflows perform similarly in the sc_mixology (Supplementary Fig. S4) and cord blood datasets (Supplementary Fig. S5), leading to an average ARI of 0.98 and 0.78, respectively, reflecting the different complexity of the two datasets. We did not observe any major differences between Leiden and Louvain clustering (Table 1).

**Fig. 6:**
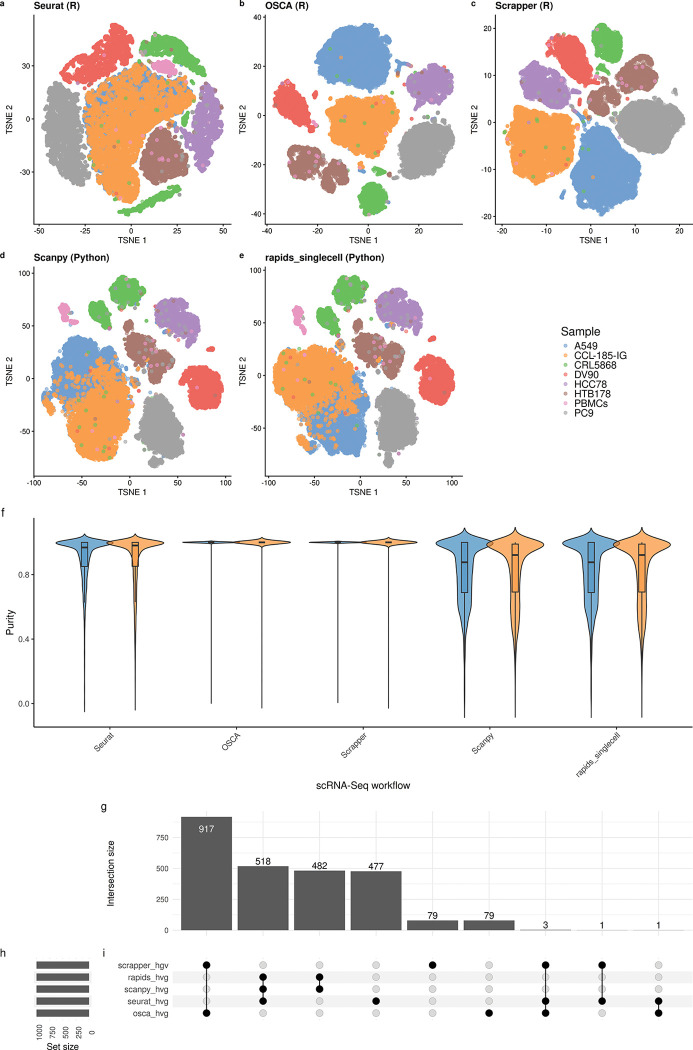
T-SNE plot and HVGs in the BE1 dataset. (a–e) t-SNE embeddings of the BE1 dataset colored by sample identity, generated using five different single-cell workflows: Seurat (a), OSCA (b), Scrapper (c), Scanpy (d), and rapids_singlecell (e). Each workflow applies its own normalization and highly variable gene (HVG) selection procedure prior to dimensionality reduction. (f–h) UpSet plot showing the intersection of HVG sets selected by each workflow. Panel (f) indicates the size of each intersection set, (g) shows the number of genes selected per method (set size), and (h) depicts the overlap structure across methods.

More interesting is the evaluation of the pipelines on the BE1 dataset, in which OSCA and scrapper achieve an almost perfect concordance with the ground truth, with an ARI of 0.95–0.97, while the other methods stop shy of 0.7 (Table 1). The main reason for this difference is the failure to separate two closely related cell lines, A549 and CCL-185-IG, which is derived from A549 cells [43]. From the t-SNE representations it is evident that OSCA and scrapper separate the two cell lines in two distinct clusters, while Seurat, Scanpy and RAPIDS achieve only partial separation (Fig. 6a–e).

To quantify the degree of separation between the two cell types and to ensure that this is not an artifact of the t-SNE representation, we computed the cell type purity in the space of the 50 PCs for each method (Fig. 6f and Supplementary Fig. S6) and the cumulative percentage of variance explained by the cell lines for each PC for each method (Supplementary Fig. S7; see Methods). These analyses confirmed that OSCA and scrapper are better able to separate these two closely-related cell lines.

We next explored the reason why this happens, by exploring the individual steps of the workflows, including QC, normalization, and clustering. Interestingly, the main reason for the difference in performance is the selection of the HGVs. Indeed, each workflow uses different ways to account for the mean-variance relation of the data in selecting HVGs (see Methods), leading to a unique set of genes for each method (Fig. 6g). The OSCA and scrapper approach to model the gene-variance trend seem to achieve greater performance than the methods employed by the other workflows (see also Supplementary Figure S6 and Supplementary Table S18).

Taken together, these results show how the algorithmic choices of the analysis workflows can impact the downstream results and how the selection of HVGs is a critical step in the pipeline.

## 3 Discussion

The ever-increasing size of single-cell datasets and the increasing availability of single-cell atlases has rendered the scalability of workflows, both in terms of speed and memory usage, crucial. Here we compared five single-cell workflows, including the popular Seurat and Scanpy packages, as well as more scalable alternatives, in terms of accuracy and scalability. Focusing on PCA as an exemplar step, we demonstrated that the type of input, the choice of algorithm, and the software configuration all critically influence the scalability of the methods.

Accelerating single-cell analyses with GPUs is a promising avenue for the near future: while GPUs are routinely used in deep learning, also in the context of single-cell analyses [45], their use to accelerate matrix multiplications and other common steps in the analysis workflows is still not widespread. One notable exception is the RAPIDS single-cell library, that we proved accurate and scalable in terms of both time and memory usage. In fact, their use in PCA calculations results in a speed up of approximately 15X compared to the fastest CPU alternative. More work is needed to make it easy for R/Bioconductor developers to leverage GPU computations in their packages: the GPUMatrix CRAN package [46] seems to be a promising starting point to achieve this goal.

One limitation of GPU is the amount of VRAM available: while the datasets used in this benchmark were small enough to fit in VRAM, for larger datasets the amount of memory needed is a critical factor for the use of GPU, as larger-than-VRAM datasets may lead to out-of-memory errors or result in slower performance due to data transfer between RAM and VRAM.

BLAS/LAPACK optimization has, perhaps not surprisingly, a profound impact on the computational performance of the methods. Indeed the same exact R code can be sped up by 15X by simply linking to an optimized BLAS/LAPACK instead of using the default reference implementation. In this benchmark we have decided to keep the default BLAS/LAPACK version in R and Python to mimic the experience of the typical user, who will likely keep the default R installation, perhaps not even realizing that such an important performance gain can be achieved with a different BLAS/LAPACK implementation. Nonetheless, our recommendation is to run R with an optimized BLAS/LAPACK and we provide instructions to do so in Ubuntu with a simple *apt* call (see Methods).

Finally, we have chosen to benchmark the most basic workflow available from each of the software frameworks, mimicking the “basic tutorials” or “getting started” guides. Obviously, modern single-cell RNA-seq studies require more complex analyses, involving multi-sample comparisons, batch-effect removal [47, 48], reference-based annotation [49], and multi-modal integration [50], to name a few. It is likely that the impact of optimized code and GPU acceleration is even more important for these more complex algorithms.

## 4 Methods

### 4.1 An introduction to PCA

Here, we briefly describe PCA and its relation to SVD. We refer the reader to [22] and [23] for a more thorough introduction and the mathematical details.

Let X be the n×m matrix that contains the log-transformed, normalized expression of m genes in n cells. We assume that the matrix X has been centered such that each row has mean 0.

The principal components are the eigenvectors of the covariance matrix and can be computed by its eigendecomposition. Alternatively, the PCs can be computed directly by the Singular Value Decomposition (SVD) of the original matrix X. Specifically, we construct the following decomposition of X:

(1) $$X=UDV^{⊤},$$

where U is a n×p orthogonal matrix of left singular vectors, V is a m×p orthogonal matrix of right singular vectors, and D is a p×p diagonal matrix, whose elements $d_{1}\geq d_{2}\geq \ldots \geq d_{p}\geq 0$ are the singular values. The first columns of UD correspond to the principal components, ordered by decreasing explained variance.

### 4.2 Algorithms to compute the SVD

#### 4.2.1 Exact algorithm

In R, an exact SVD algorithm is implemented in the base svd function, which is called by the *BiocSingular* package’s runPCA function when selecting the “exact” method. In Python, *scikit-learn*’s PCA function calls the scipy.linalg.svd function. Both R’s and Python’s svd functions internally call the gesdd funcion in the LAPACK (Linear Algebra Package) library in Fortran. This function implements an efficient divide-and-conquer algorithm to compute the SVD [51].

#### 4.2.2 Truncated SVD

Often, one is interested in computing only a few, say k, singular values and vectors. For instance, in scRNA-seq, we are often limiting ourselves in calculating the top 30 or 50 PCs. Specifically, we may solve the problem:

$$X^{\left(k\right)}=U_{k}D_{k}V_{k}^{⊤},$$

where $U_{k}$ and $V_{k}$ denote the leading k columns of U and V, respectively, $D_{k}$ denotes the diagonal matrix with the k largest singular values. It can be shown that $X^{\left(k\right)}$ is the best rank-k approximation of X.

When k≪min{n,m}, as in the case of scRNA-seq analysis, truncated SVD algorithms exist to efficiently calculate the k largest singular values and the corresponding singular vectors. Here, we review a few of these methods.

##### ARPACK

ARPACK [28] is a collection of Fortran77 subroutines designed to solve large-scale eigenvalue problems. ARPACK stands for ARnoldi PACKage and is based on the Arnoldi iteration method [52]. This method provides a good approximation of the k largest singular values and the corresponding singular vector of a matrix by constructing an orthonormal basis of its Krylov subspace [52]. Thanks to the relation between eigendecomposition and SVD, the ARPACK algorithm can be used to efficiently calculate the k largest singular values and the corresponding singular vectors.

Consider the decomposition in (1), one can show that

$$X^{⊤}X=VD^{2}V^{⊤},$$

and that $U=XVD^{-1}$. Thus, the singular values and singular vectors of X can be computed from the eigenvalues and eigenvectors of $X^{⊤}X$.

The Arnoldi method is particularly useful when dealing with large sparse matrices, as the algorithm does not require explicitly the whole matrix, but only a matrix-vector multiplication result. Therefore, if the matrix-vector product can be computed efficiently, which is the case when X is sparse, ARPACK will be very efficient for large-scale matrices.

The ARPACK Fortran routine is used by Scanpy’s PCA function through Scipy’s svds function. In R, the *RSpectra* function provides an interface to the Spectra C++ library [35] that reimplements the ARPACK algorithms. Since Spectra implements the same underlying algorithm, we refer to both methods as ARPACK in the main text.

##### Random SVD

The randomized SVD algorithm [29] computes a near-best rank k approximation of a matrix using matrix-vector products with standard Gaussian vectors. The randomized SVD algorithm uses a random projection matrix to sample the column space of the original matrix, allowing approximation of the SVD of the original matrix by computing SVD on a smaller matrix.

In detail, given a n×l orthonormal matrix Q, with k≤l≤m, such that $X\approx QQ^{⊤}X$:

- Form $B=Q^{⊤}X$;
- Compute the SVD of B, i.e., $B=\tilde{U}\Sigma V^{⊤}$;
- Set $U=Q\tilde{U}$.

Since $X\approx QQ^{⊤}X=Q\left(\tilde{U}\Sigma V^{⊤}\right)$, it follows that $X\approx U\Sigma V^{⊤}$. This algorithm is efficient when l≪m, because we can efficiently compute the matrix B and then we just need to compute the SVD of a much smaller matrix.

The randomness comes from the construction of the orthonormal matrix Q, which is obtained by concatenating l randomly generated Gaussian random vectors [29].

The randomized SVD algorithm is implemented in the *rsvd* R package, internally called by *BiocSingular* and in the truncatedSVD function in *scikit-learn*, which is called by Scanpy.

##### IRLBA

The Augmented Implicitly Restarted Lanczos Bidiagonalization Algorithm (IRLBA) [31] is a fast and memory-efficient method for computing truncated SVD of a matrix. It is based on the augmented implicitly restarted Lanczos bidiagonalization algorithm, which is a variant of the Lanczos algorithm that uses a bidiagonalization step to reduce the dimensionality of the matrix.

IRLBA computes sequences of projections of X onto specific low-dimensional subspaces that reduce the dimensionality of the problem. Restarting is implemented to iteratively reduce the approximation error.

More specifically, the algorithm applies l steps of partial Lanczos bidiagonalization to X and yields the decomposition

XP=QB,

where P and Q are orthonormal matrices of dimension m×l and n×l, respectively, while B is upper bidiagonal. An approximate SVD of X can be obtained starting from the SVD of B and the matrices P and Q [31].

When X is large, the storage requirement of the partial Lanczos bidiagonalization is large, unless the number of Lanczos bidiagonalization steps is small. However, for a small value of l, the SVD of X may be approximated poorly by the algorithm. To avoid this problem, the algorithm uses a sequence of initial vectors to form P, a technique known as restarted partial Lanczos bidiagonalization. Finally, to avoid numerical instabilities, restarting is carried out by augmentation of Krylov subspaces (see [31] for details).

The IRLBA algorithm is implemented in the *irlba* R package, internally called by *BiocSingular*.

#### 4.2.3 IPCA

Incremental PCA [33] is a method for computing the principal components of a matrix in an incremental manner. This means that the algorithm processes the data in batches, computing the principal components for each batch and then combining them to obtain the final result. This approach avoids the need to load the entire dataset into memory, making it suitable for large-scale data analysis. The basic idea is to update the principal components incrementally as new data batches become available.

Here, we briefly illustrate the algorithm, assuming two batches of data. Let us denote with $X_{A}$ and $X_{B}$ the two batches of data. For the first batch, we compute the SVD in the usual way, say, $X_{A}=U_{A}D_{A}V_{A}^{⊤}$. The goal is to find the SVD of the concatenation of $X_{A}$ and $X_{B}$, which can be expressed as

$$\left[X_{A}X_{B}\right]=\left([U_{A}{\tilde{X}}_{B}]\tilde{U}\right)\tilde{D}\left({\tilde{V}}^{⊤}\left[\begin{array}{cc} V_{A}^{⊤} & 0\\ 0 & I \end{array}\right]\right),$$

where ${\tilde{X}}_{B}$ is the component of $X_{B}$ orthogonal to $U_{A}$ and $\tilde{R}=\tilde{U}\tilde{D}{\tilde{V}}^{⊤}$ is the SVD of a matrix of size k+d, with d the number of observation in the batch $X_{B}$, which can be computed efficiently even for large n. The algorithm is slightly more complicated because it needs to take into account the fact that the sample mean of the data will change as more batches are added, the full details are in [33].

The IPCA algorithm is implemented in the IncrementalPCA function in *scikit-learn*.

#### 4.2.4 Jacobi

The Jacobi SVD algorithm [32] is an iterative algorithm that applies a sequence of plane rotations to a symmetric matrix to bring it to diagonal form. The Jacobi SVD algorithm is particularly known for its numerical stability and accuracy, making it suitable for high-precision applications.

The Jacobi method is an algorithm for the diagonalization of symmetric matrices and can be used to compute the SVD of general matrices. Indeed, applying the Jacobi method to $H=XX^{⊤}$ leads to the sequence $A^{\left(k+1\right)}=A^{\left(k\right)}V^{\left(k\right)}$, whose limit matrix is UD, while the accumulated product of Jacobi rotations is V [32].

The Jacobi algorithm is implemented in the *RAPIDS* library.

### 4.3 SVD Performance Evaluation

#### 4.3.1 SVD Accuracy

To evaluate the degree of similarity among the principal components obtained from different SVD algorithms, we computed the absolute Pearson correlation coefficient between the PC computed with the “exact” method and the corresponding PC derived from all other methods. The Pearson correlation coefficient measures the linear correlation between two variables, ranging from −1 to 1, where values close to zero indicate weak or no linear correlation, and values close to one indicate a strong linear correlation. By computing the absolute value of the correlation we account for PCA’s invariance with respect to rotations. High values of absolute correlation coefficients indicate high similarity in the patterns captured by the principal components.

#### 4.3.2 Computational Time

To measure the computational time for PCA computation in R, we employed the proc.time function, capturing the start and end times for each computational step. Conversely, in Python, we imported the time module and used the time.time function to capture the start time of the process, then using the sys.argv command we calculate the end time of the process.

#### 4.3.3 Memory Usage

To monitor memory usage efficiently, we employed different strategies in R and Python. In R, we used the Rprof tool to profile memory consumption during program execution. With Rprof, we were able to track the memory consumption and then identify the peak of memory usage. Conversely, in Python, we used the /usr/bin/time -v command within our Slurm job scripts to measure and record the maximum memory usage. By appending this command to our job scripts, we captured memory usage statistics at the process level, allowing us to monitor and analyze resource utilization effectively.

#### 4.3.4 Ranking SVD methods

The Plackett-Luce model [41, 42] provides a flexible framework for modeling preferences and rankings in various domains. It handles ties of arbitrary order in the ranking. This means that the model can accommodate rankings in which items are tied for a particular rank. To model such rankings, we utilized the *PlackettLuce* R package, which extends the traditional Plackett-Luce model by incorporating methods that accommodate tied rankings. Using the as.rankings function, the data were restructured into a format suitable for analysis by converting preferences into rankings. The functions used included the weights argument, which allows for the weighting of observations based on the frequency of preferences, and the log = FALSE option, which transforms the scores into normalized probabilities, ensuring that their sum equals one. This step is crucial, as the first item in the ranking is treated as the reference for others, allowing the calculation of relative probabilities for each item to be placed in the top position.

Subsequently, we performed a summary analysis of the results using the summary function, which provided detailed information on the estimated coefficients of the model. To visualize the relative importance of each method, we used the qvcalc function, which calculates the worth values for each method, enabling the graphical representation of the results.

### 4.4 A typical workflow for Single Cell RNA-seq data analyses

Here, we describe the steps of a typical workflow for single-cell RNA-seq (scRNA-seq) data. These steps are common to all five pipelines that we benchmarked in this paper. After a general description of all the steps, we describe how each specific workflow differ in the implementations of these steps.

#### 4.4.1 Preprocessing

Low-quality libraries in scRNA-seq data can result from various sources, such as cell damage during dissociation or errors in library preparation (e.g., inefficient reverse transcription or PCR amplification). These typically manifest as “cells” with low total counts, few expressed genes, and high proportions of mitochondrial reads, leading to misleading results in downstream analyses [53].

After excluding empty droplets and identifying potential doublets, droplets containing damaged cells or low-quality reads are filtered out. Droplets are small compartments that capture single cells and barcoded beads during scRNA-seq experiments. Empty droplets lack cells, while doublets occur when two cells are mistakenly captured in the same droplet, creating mixed signals that can compromise data accuracy.

The library size, defined as the total sum of counts across all relevant features for each cell, is a commonly used metric for filtering. Cells with small library sizes are more likely to be of low quality due to RNA loss during library preparation, either from cell lysis or inefficient cDNA capture and amplification. Another metric is the number of expressed features per cell, defined as the number of endogenous genes with non-zero counts for that cell. Cells with very few expressed genes are likely of poor quality as the diverse transcript population has not been successfully captured. The proportion of reads mapped to mitochondrial genes can also be used; high proportions suggest possible loss of cytoplasmic RNA due to cell damage, as mitochondria, being larger than individual transcript molecules, are less likely to escape through cell membrane holes.

#### 4.4.2 Normalization

Normalization aims to adjust raw counts for variable sampling effects by scaling the observed counts. This process helps to reduce technical noise and preserve biological variation. Systematic differences in coverage between libraries in scRNA-seq data, often due to variations in cDNA capture or PCR amplification efficiency, can interfere with expression profile comparisons. Normalization removes these differences, ensuring accurate clustering and differential expression analyses [54].

#### 4.4.3 Feature selection

Feature selection aims at reducing the dimension of the input matrix to identify genes whose expression is variable enough across cells to be able to capture most of the cell heterogeneity filtering out noise. Methods such as dimensionality reduction and clustering are generally performed using the filtered matrix [55–57].

#### 4.4.4 Dimensionality Reduction: PCA, t-SNE, UMAP

Dimensionality reduction techniques are typically used for the visualization of complex datasets. These methods aim to transform high-dimensional data into a lower-dimensional space preserving the underlying structure and variability inherent in the data.

Principal Component Analysis (PCA) is widely employed in scRNA-seq to identify orthogonal axes (principal components) that capture the maximum variance in the dataset. By projecting cells onto these components, PCA provides a simplified representation that retains the most significant sources of variation.

In contrast, t-Distributed Stochastic Neighbor Embedding (t-SNE) [58] focuses on preserving local similarities between cells in the high-dimensional space. It maps cells into a low-dimensional space (often 2D) that aims at placing similar cells together. Uniform Manifold Approximation and Projection (UMAP) [59] is a more computationally efficient alternative that has gained popularity in recent years.

It is a standard practice that both t-SNE and UMAP take as input the first 50 PCs obtained from the pre-processed gene expression matrix, where 50 is a number of PCs that should guarantee to capture most of the cell heterogeneity present in the data.

#### 4.4.5 Clustering: Louvain, Leiden

Clustering approaches such as Louvain [60] and Leiden [61] are used to identify discrete cell populations based on gene expression patterns.

These methods operate on a graph representation of the data, often constructed using k-nearest neighbors (KNN) or shared nearest neighbors (SNN) algorithm, where nodes represent cells, and edges represent similarities or connections based on gene expression profiles.

An important concept to this clustering approaches is modularity, which is used to evaluate and optimize the grouping of cells into meaningful clusters, also referred to as communities. Modularity is a measure used to evaluate how well cells are grouped into clusters based on their gene expression profiles. It quantifies the quality of clustering by comparing the density of connections (or similarities) between cells within the same cluster to those between cells in different clusters. Higher modularity indicates that cells within a cluster are more tightly connected (e.g., share similar gene expression patterns) compared to connections between clusters. Lower modularity implies that the boundaries between clusters are less distinct, potentially reflecting noise or suboptimal clustering. In the single-cell RNA-seq context, communities refer to groups of cells that are more connected or similar to one another based on similarity metrics, such as shared gene expression patterns, than they are to cells in other groups. These communities may represent biologically meaningful structures, such as cell types, subtypes, or states, within scRNA-seq datasets.

### 4.5 Workflows for Single Cell RNA-seq data analyses

#### 4.5.1 Seurat

Seurat, developed and maintained by the Satija Lab, is an R toolkit tailored for single-cell genomics. For our analysis, we utilized Seurat version 5 [19].

Specific steps and default values of the Seurat workflow include:

- Find Mitochondrial Genes: This step identifies mitochondrial genes by calculating the proportion of transcripts mapping to mitochondrial genes, using the PercentageFeatureSet function with the regex pattern “^MT-”or “^mt-” from *Seurat*. Cells with high mitochondrial content (less than 5 %) or low total counts are flagged and filtered out.
- Filtering: A filtering step is applied to remove cells and genes with undesired properties. To do the subset is used the subset function from *SeuratObject*. Cells are retained if they express between 200 and 5000 features, have mitochondrial content below 5%, and a total count under 25,000 for BE1 dataset. Cells are retained if they express between 200 and 2500 features, have mitochondrial content below 5%, and a total count under 4,000 for cord blood dataset. Cells are retained if they express between 200 and 6200 features, have mitochondrial content below 5%, and a total count under 60,000 for sc_mixology dataset.
- Normalization: The data is normalized using the LogNormalize method, which scales gene expression counts by the total expression for each cell, followed by a log transformation to stabilize variance.
- Highly Variable Genes: Highly variable genes are identified using the variance-stabilizing transformation (vst) method, with the top 1,000 features selected. The function used is FindVariableFeatures from *Seurat*
- Scaling: All genes are scaled using the ScaleData function, which centers the expression values around zero and normalizes variance.
- PCA: Principal Component Analysis (PCA) is conducted using the highly variable genes and retaining the first 50 PCs. The algorithm used is IRLBA.
- t-SNE: RunTSNE from *Seurat* is applied on the first 50 PCs. The perplexity parameter is set to 18.
- UMAP: The runUMAP function of the Seurat package reduces dimensionality starting from the top 50 PCs using the *uwot* R package implementation, using the cosine metric.
- Louvain: FindNeighbors function, a Shared Nearest Neighbor (SNN) Graph is constructed setting k equal to 20. FindClusters function is applied setting *algorithm* parameter equal to 1 for Louvain algorithm.. The Louvain algorithm identifies clusters by constructing a k-nearest neighbors graph (based on the top 50 components) and optimizing modularity. Clusters are generated with a resolution of 0.2 for BE1, 0.1 for sc_mixology, 0.2 for cord blood.
- Leiden: FindClusters function is applied setting *algorithm* parameter equal to 4 for Leiden algorithm. The Leiden algorithm identifies clusters by constructing a k-nearest neighbors graph (based on the top 50 components) and optimizing modularity. Clusters are generated with a resolution of 0.2 for BE1, 0.08 for sc_mixology, 0.2 for cord blood.

#### 4.5.2 Orchestrating Single-Cell Analysis (OSCA)

Orchestrating Single-Cell Analysis (OSCA) with Bioconductor is a framework and set of tools within the Bioconductor ecosystem for analyzing scRNA-seq data. It focuses on integrating multiple R packages to provide a comprehensive workflow for processing, analyzing, and visualizing single-cell datasets. Bioconductor uses the SingleCellExperiment class for storing single-cell assay data and metadata: count matrices are stored in the assays component, where rows represent features (e.g. genes, transcripts) and columns represent cells. In addition, low-dimensional representations of the primary data, and metadata describing cell or feature characteristics can also be stored in the SingleCellExperiment object. By standardizing the storage of single-cell data and results, Bioconductor supports interoperability between single-cell analysis packages.

Specific steps and default values of the OSCA workflow include:

- Find Mitochondrial Genes: Mitochondrial genes are identified by mapping gene symbols to their chromosomal location using the *EnsDb.Hsapiens.v75* package for human datasets and the *EnsDb.Mmusculus.v79* package for mouse datasets. Metrics such as mitochondrial percentage and total counts per cell are calculated using the perCellQCMetrics function from *scuttle*. Cells with high mitochondrial content (less than 5%) or low total counts are flagged and filtered out.
- Filtering: Cells are filtered based on sequencing depth and quality control metrics obtained in the previous step.
- Normalization: The logNormCounts function of the *scuttle* package normalizes gene expression data by calculating size factors and performing log-transformation.
- Highly Variable Genes: The modelGeneVar function of the *scran* Bioconductor package models gene expression variance across cells, and the getTopHVGs function selects the top 1,000 most variable genes.
- Scaling: In the OSCA suggestion there isn’t a specific function for running the scaling step. In this case, we set scale = TRUE in the runPCA function to compute the scaling.
- PCA: The runPCA function of the *scater* package reduces dimensionality using the previously identified HVGs, by default using the Exact algorithm from runSVD in *BioCSingular* package and computing the first 50 PCs. The runPCA function performs scaling as part of the PCA computation.
- t-SNE: The runTSNE function of the *scater* package reduces dimensionality starting from the top 50 PCs. By default, the function will set a perplexity that scales with the number of cells.
- UMAP: The runUMAP function of the *scater* package reduces dimensionality starting from the top 50 PCs.
- Louvain: The clusterCells function from *scran*, with the NNGraphParam parameter, applies the Louvain algorithm to cluster cells based on the top 50 PCs and using a resolution of 0.5 for all the datasets. This function is a wrapper around clusterRows function from the *bluster* package.
- Leiden: The clusterCells function, configured with the NNGraphParam parameter, applies the Leiden algorithm to cluster cells based on the top 50 PCs and using a resolution of 0.5 for all the datasets.

#### 4.5.3 scrapper

The Bioconductor package *scrapper* reimplements some of the OSCA functions in C++. It provides R bindings to C++ code for the analysis of single-cell expression data, primarily utilizing various *libscran* libraries. Each function within the workflow addresses a specific step in the single-cell analysis pipeline, including tasks such as quality control, clustering, and marker detection.

Specific steps and default values of the scrapper workflow include:

- Find Mitochondrial Genes: Mitochondrial genes are identified by applying a regular expression pattern “^mt-” or “^MT-” to the row names of the count matrix. This step allowed for the selection of genes associated with mitochondrial function.
- Filtering: RNA quality control metrics are computed using the computeRnaQcMetrics function, which included subsets for mitochondrial genes. Suggested thresholds for filtering were determined using the suggestRnaQcThresholds function. The filterRnaQcMetrics function was then applied to retain cells that met the established quality criteria.
- Normalization: The filtered count matrix was log-normalized using size factors derived from the RNA quality metrics using centerSizeFactors. This log-normalization was performed using the normalizeCounts function, ensuring that the data were adjusted for sequencing depth and other technical variations.
- Highly Variable Genes: Gene variances were modeled using the modelGeneVariances function. Highly variable genes were identified using the chooseHighlyVariableGenes function, selecting the top 1000 genes based on their variability.
- Scaling: In this package there isn’t a specific function for running the scaling step. In this case, we set scale = TRUE in the runPca function to compute the scaling.
- PCA: PCA was conducted on the normalized data of the highly variable genes using the runPca function, which by default computes the first 25 PCs. To keep the PCA results comparable, we decided to compute the first 50 PCs.
- t-SNE: t-SNE was applied to the fist 50 PCs and a default perplexity of 30. This was executed using the runTsne function.
- UMAP: UMAP was performed on the first 50 PCs, and the default number of neighbors set to 15.
- Louvain: A shared nearest neighbor (SNN) graph was constructed from the top 50 PCs using the buildSnnGraph function. The Louvain algorithm (Multilevel) was applied to this graph with a resolution of 0.18 for BE1 dataset, 0.16 for sc_mixology, 0.20 for cord blood.
- Leiden: A shared nearest neighbor (SNN) graph was constructed from the top 50 PCs using the buildSnnGraph function. The Leiden algorithm was applied to this graph with a resolution of 0.18 for BE1 dataset, 0.16 for sc_mixology, 0.20 for cord blood.

#### 4.5.4 Scanpy

Scanpy [18] is a scalable Python package for analyzing single-cell gene expression data, encompassing a wide array of functionalities such as preprocessing, visualization, clustering, pseudotime and trajectory inference, differential expression testing, and simulation of gene regulatory networks.

Specific steps and default values of the Scanpy workflow include:

- Find Mitochondrial Genes: This step identifies mitochondrial genes by annotating those whose names start with “MT-” or “mt-”. Quality control (QC) metrics, including the percentage of mitochondrial counts per cell, are calculated using sc.pp.calculate_qc_metrics
- Filtering: A filtering step is applied to remove cells and genes with undesired characteristics using scanpy.pp.filter_cells and scanpy.pp.filter_genes functions in *scanpy* package. For BE1 and sc_mixology dataset cells expressing fewer than 200 genes and genes present in fewer than 3 cells are excluded. Additionally, cells with an excessive number of expressed genes (> 5000) or a high percentage of mitochondrial counts (> 5%) are filtered out. For cord blood dataset cells expressing fewer than 200 genes and genes present in fewer than 3 cells are excluded. Additionally, cells with an excessive number of expressed genes (> 2500) or a high percentage of mitochondrial counts (> 5%) are filtered out.
- Normalization: scanpy.pp.normalize_total function normalizes each cell by total counts over all genes. scanpy.pp.log1p perform log-transformation on the normalized gene expression.
- Highly Variable Genes: Highly variable genes are identified using sc.pp.highly_variable_genes function using Seurat method. Specific thresholds for mean expression (min_mean=0.0125, max_mean=3) and dispersion (min_disp=0.5). Up to 1,000 highly variable genes are selected for downstream analyses.
- Scaling: All genes are scaled using scanpy.pp.scale function, data is scaled to center values around zero and normalize variance, with a maximum absolute value of ±10.
- PCA: Principal Component Analysis (PCA) is performed using the Arpack SVD solver and retaining the first 50 PCs. The function used is scanpy.tl.pca
- t-SNE: t-SNE was applied to the first 50 PCs and a default perplexity of 30. This was executed using the scanpy.tl.tsne function.
- UMAP: UMAP is applied to the first 50 PCs and the number of neighbors is set by default to 10. The function used is scanpy.tl.umap.
- Louvain: The nearest neighbors distance matrix and a neighborhood graph of observations is computed with scanpy.pp.neighbors function. The size of local neighborhood (in terms of number of neighboring data points) used for manifold approximation is 10 on the top 50 PCs. The Louvain clustering algorithm is applied with scanpy.tl.louvain function and a resolution of 0.13 for BE1 dataset, sc_mixology and for cord blood.
- Leiden: Similar to Louvain, the Leiden algorithm performs clustering using this scanpy.tl.leiden function with a resolution of 0.13 for BE1 dataset, sc_mixology and for cord blood.

#### 4.5.5 rapids_singlecell

rapids_singlecell [62] is a Python-based workflow that utilizes GPU computing to enhance the analysis of single-cell data. This workflow is integrated within the *scverse* environment, and its structure is built upon *Scanpy*. By leveraging GPU capabilities through CuPy and NVIDIA’s RAPIDS framework, this approach prioritizes computational efficiency, facilitating the rapid analysis of large-scale single-cell datasets.

Specific steps and default values of the rapids_singlecell workflow include:

- Find Mitochondrial Genes: Mitochondrial (MT) and ribosomal (RIBO) genes are identified and annotated based on their respective prefixes (“MT-” and “RPS”) using *rsc.pp.flag_genefamily*. Quality control (QC) metrics, including percentages of MT and RIBO counts, are calculated to assess sequencing quality using *rsc.pp.calculate_geneqc_genemetrics* function.
- Filtering: A filtering step is applied to remove cells and genes with undesired characteristics using rsc.pp.filter_cells and rsc.pp.filter_genes functions in *rapids_singlecell* package. For BE1, sc dataset cells expressing fewer than 200 genes and genes present in fewer than 3 cells are excluded. Additionally, cells with an excessive number of expressed genes (> 5000) or a high percentage of mitochondrial counts (> 5%) are filtered out.
- Normalization: rsc.pp.normalize_total function normalizes each cell by total counts over all genes. rsc.pp.log1p perform log-transformation on the normalized gene expression.
- Highly Variable Genes: Highly variable genes are identified using the Seurat v3 method with n_top_genes=1000 and the counts layer.
- Scaling: Data is scaled to zero mean and unit variance, with a maximum absolute value of plus/minus 10 to prevent extreme values from dominating the analysis.
- PCA: PCA is performed using 50 components and the auto algorithm could be one between Exact or Jacobi.
- t-SNE: t-SNE is applied to the first 50 PCs and with a perplexity of 30. *rapids_singlecell.tl.tsne* is used to perform t-SNE.
- UMAP: UMAP is applied to the first 50 PCs and the number of neighbors is set by default to 10. *rapids_singlecell.tl.umap* is used to perform umap.
- Louvain: the nearest neighbors distance matrix and a neighborhood graph of observations is computed with *rapids_singlecell.pp.neighbors* function. he size of local neighborhood (in terms of number of neighboring data points) used for manifold approximation is 10 on the top 50 PCs. The Louvain clustering algorithm is applied with rapids_singlecell.tl.louvain function and a resolution of 0.6 for BE1 dataset, 0.1 for sc_mixology, 0.1 for cord blood.
- Leiden: Similar to Louvain, the Leiden algorithm performs clustering using this rapids_singlecell.tl.leiden function with a resolution of 0.6 for BE1 dataset, 0.1 for sc_mixology, 0.1 for cord blood.

### 4.6 Datasets

#### 4.6.1 1.3M cell dataset (1.3M)

This dataset contains the gene expression of 1.3 million brain cells isolated from E18 mice and was generated using the 10x Genomics platform for the investigation of cellular heterogeneity within the developing mouse brain at embryonic day 18 (E18) [38]. Cells from the cortex, hippocampus, and ventricular zone of two embryonic mice were dissociated and used to create 133 scRNA-Seq libraries. The samples were sequenced on 11 Illumina Hiseq 4000 flow cells, resulting in a read-depth of approximately 18,500 reads per cell. The sequencing data were processed by Cell Ranger 1.2 to generate single-cell expression profiles of 1,308,421 cells. We refer to this dataset as the 1.3M cell dataset.

This dataset has been used for benchmarking the scalability of both PCA methods and different pipelines comparing different dataset subsampling. This dataset lacks a ground truth for the cell-type annotation.

The downsampling was performed at the cellular level using the sample function in R, selecting random subsets of 100k, 500k, and 1M cells without replacement. The total number of genes remained constant across all subsets.

#### 4.6.2 Cite-seq coord blood (cb)

CITE-seq (Cellular Indexing of Transcriptomes and Epitopes by sequencing) applied to cord blood samples provides a comprehensive and high-dimensional dataset that captures both transcriptomic and proteomic information at the single-cell level [44]. This innovative technique combines single-cell RNA sequencing with the measurement of surface protein markers, allowing for the simultaneous profiling of gene expression and cell surface protein expression within individual cells. CITE-seq data are a combination of two data types extracted simultaneously from the same cell. The first data type is scRNA-seq data, while the second one consists of up to a hundred antibody-derived tags (ADT). We analyze the transcriptomic measurements paired with abundance estimates for 11 surface proteins, whose levels are quantified with DNA-barcoded antibodies. The dataset contains 20,400 genes and 7,858 cells. Cell type annotation was performed by leveraging ADT surface protein marker data to manually gate and classify cells based on immunological knowledge of marker distributions. This manual gating was informed by established marker-to-cell type mappings, as described in [63]. These annotations were used to generate a reference set of labels independent of scRNA-seq-derived predictions, enabling robust benchmarking of scRNA-seq analysis methods.

#### 4.6.3 BE1

BE1 is a single-cell RNAseq benchmark dataset providing a controlled heterogeneity environment using lung cancer cell lines characterised by expressing seven different driver genes (EGFR, ALK, MET, ERBB2, KRAS, BRAF, ROS1) [43]. Specifically the following lung cancer cell lines are included:

- PC9, 4492 sequenced cells (EGFR Del19, activating mutation [64])
- A549, 6898 sequenced cells (KRAS p.G12S, growth and proliferation, [65])
- NCI-H596 (HTB178), 2965 sequenced cells (MET Del14 , enhanced protection from apoptosis and cellular migration [66])
- NCI-H1395 (CRL5868), 2673 sequenced cells (BRAF p.G469A, gain of function, resistant to all tested MEK ± BRAF inhibitors, [67])
- DV90, 2998 sequenced cells (ERBB2 p.V842I, increases kinase activity, [68])
- HCC78, 2748 sequenced cells (SLC34A2-ROS1 Fusion, ROS1 inhibitors have antiproliferative effect [69])
- CCL.185.IG, 6354 sequenced cells EML4-ALK Fusion-A549 Isogenic Cell.

The experiment was done using CellPlex technology from 10x Genomics allowing multiplexing samples into a single channel and therefore removing unwanted batch effects. BE1 is composed of 36,753 genes and 29,606 cells. This dataset has been used for benchmarking the pipelines and the cell line is used as the ground truth for the cell-type annotation.

#### 4.6.4 sc_mixology

Sc_mixology uses three human lung adenocarcinoma cell lines HCC827, A549, H1975, H838 and H2228, which were cultured separately and then processed in three different ways [17]. Single cells from each cell line were mixed in equal proportions, with libraries generated using three different protocols: CEL-seq2, Drop-seq (with Dolomite equipment) and 10x Chromium. In this work, we use only single cells from the mixture of five cell lines with 10x Chronium protocol (GSM3618022). Sc_mixology containing 11,786 genes and 3,918 cells. This dataset has been used for benchmarking the pipelines and the cell line is used as the ground truth for the cell-type annotation.

### 4.7 Workflow performance evaluation

#### 4.7.1 ARI

The Adjusted Rand Index (ARI) [70] is a statistical measure commonly used to assess the similarity between two clustering results. It takes into account both the agreement and disagreement between data points in the original and clustered datasets, providing a normalized index that ranges from 0 to 1. In this benchmark, we opt to employ the ARI for the comparative assessment of various pipelines comparing the known cell type annotation with that obtained after the cluster algorithms.

#### 4.7.2 Cell type purity

Cell type purity is defined as the percentage of neighbors for each cell that belong to the same cell type. Well-separated cell types should exhibit high purity as the cells from different types do not mix. Low purity corresponds to a data representation in which cells of different types are mixed together. We computed the cell type purity using the function neighborPurity from the *bluster* Bioconductor package [71], using the known cell types as “clusters” and the default value of k=50 nearest neighbors.

#### 4.7.3 Variability explained by cell types

To quantify the proportion of total variability explained by the cell types, we employed a strategy inspired from the *scDiagnostics* Bioconductor package [72]. Briefly, for each method and each PC, we multiplied the percentage of variance explained by each PC by the $R^{2}$ index of a linear model that uses that PC as a response variable and the cell type as covariate.

### 4.8 Infrastructure

#### 4.8.1 PCA

The computational resources employed for the generation of the results presented in this research paper consisted of a dual-processor configuration featuring 2x AMD EPYC 7301 CPUs clocked at 2.7 GHz, specifically provided by DiBio UniPD. Additionally, substantial computational acceleration was achieved through the utilization of four NVIDIA A100 SXM4 GPUs, each equipped with 40GB of memory and integrated into the JetStream platform. These high-performance computing resources played a pivotal role in facilitating the execution of complex calculations and simulations, thereby contributing to the robustness and accuracy of the findings presented in this study.

#### 4.8.2 Pipeline Single Cell

In the pursuit of benchmarking the single-cell pipelines presented in this research paper, computational resources from the CAPRI facility at the University of Padova were leveraged. The CPU component of this infrastructure was comprised of 16 Intel(R) Xeon(R) Gold 6130 processors, each operating at a clock speed of 2.10GHz (CAPRI UniPD). Additionally, we utilized 2 NVIDIA Tesla P100 GPUs for GPU-intensive tasks, each equipped with 16GB of memory (CAPRI UniPD). These resources, available through CAPRI, played a crucial role in executing the computational workflows associated with the benchmarking analyses, ensuring the efficiency and accuracy of the results detailed in this study.

#### 4.8.3 BLAS/LAPACK version

To optimize the performance of linear algebra operations in R, the default Ubuntu BLAS/LAPACK implementation can be replaced with the OpenBLAS library, which provides substantial computational improvements. On Ubuntu systems, this configuration can be readily modified using the apt package manager. Initially, the desired OpenBLAS version must be installed using sudo apt install libopenblas0-pthread for the parallelized version, or libopenblas0-serial for the single-threaded implementation. Subsequently, the update-alternatives command is employed to register the new library as an available alternative, specifying a priority level (e.g., 110 for the pthread version). The system administrator can then interactively select the desired implementation through sudo update-alternatives --config. This procedure must be repeated for both BLAS and LAPACK libraries, replacing the libblas.so.3 and liblapack.so.3 files respectively. Proper configuration can be verified by executing sessionInfo() in R, which will display the path of the currently utilized library. This approach enables seamless transitions between different BLAS implementations without requiring R recompilation, thereby allowing researchers to select the configuration most suitable for their specific computational requirements.

#### 4.8.4 Code availability

The code to reproduce all the analysis described in this paper, including the benchmark of SVD methods and scRNA-seq workflows is available at https://github.com/billila/pca_scwf_paper. In the same repository, we provide definition files for the containers to reproduce all the results. The integration of the ARPACK algorithm into the BiocSingular package is available at https://github.com/billila/BiocSingular. The package versions and software dependencies used in this study are provided in the definition files available at https://github.com/billila/pca_scwfpaper/envs.

#### 4.8.5 Data availability

The 1.3M cell dataset is available as part of the TENxBrainData Bioconductor package at https://bioconductor.org/packages/TENxBrainData. sc_mixology dataset is available at https://ncbi.nlm.nih.gov/geo/query/acc.cgi?acc=GSE118767 and more tecnichal details at https://github.com/LuyiTian/sc_mixology. The BE1 dataset is available at https://doi.org/10.6084/m9.figshare.23939481.v1. The cord blood dataset is available as part of the *SingleCellMultiModal* package at https://bioconductor.org/packages/SingleCellMultiModal

## Supplementary Material

Supplement 1

## Figures and Tables

**Table 1:** Adjusted Rand Index (ARI) between the workflow and between single-cell RNA-seq dataset.

ARI	Algorithm	Seurat	OSCA	Scanpy	Rapids	scrapper

cb	louvain	0.88	0.81	0.75	0.78	0.80
	leiden	0.7	0.81	0.75	0.77	0.81

sc_mix	louvain	0.97	0.96	0.99	0.99	0.96
	leiden	0.98	0.96	0.99	0.99	0.96

BE1	louvain	0.67	0.97	0.68	0.68	0.97
	leiden	0.68	0.95	0.68	0.68	0.97
