# Supplementary material for: Benchmarking large-scale single-cell RNA-seq analysis
Source: bioRxiv. 2025 Oct 29:2025.10.28.681564. Preprint. [Version 1] doi: 10.1101/2025.10.28.681564 (PMC12636554; doi:10.1101/2025.10.28.681564)
Supplement: Supplement 1 [file media-1.pdf]

Supplementary Material for "Benchmarking large-scale single-cell RNA-seq analysis"

October 28, 2025

1 Supplementary Figures and Tables

	Number of Genes	Number of Cells	Species	Sample	Technology	Ground Truth
**1.3M**	27,998	1,306,127	Mouse	Brain	10X Genomics Chromium Protocol	No
**BE1**	36,753	29,606	Human	Lung Adenocarcinoma	10X Genomics Chromium Protocol	Lung Cancer Cell Lines
**cb**	20,400	7,858	Mouse	Cord Blood Mononuclear	CITE-seq	Manual Gating Antibody Expression Reference
**sc_mixology**	11,786	3,918	Human	Lung Adenocarcinoma	10X Genomics Chromium Protocol	Lung Cancer Cell Lines

Table S1: Summary of datasets used to compare the four workflows of single-cell analysis.

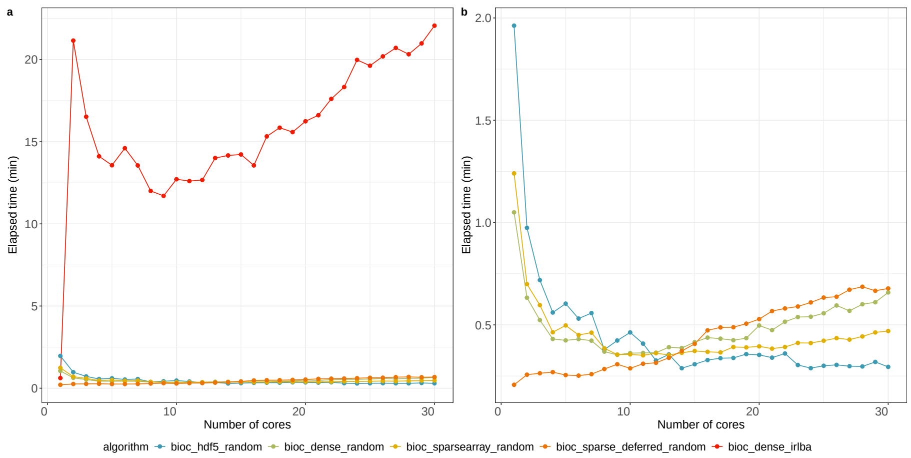

Figure S1: a. Elapsed time for increasing number of cores for best performing method for 100k in R. b. Same as a. without *bioc_dense_irlba*.

**Method's Name**	**SVD Algorithm**	**Mat type**	**Library**	**software**	**CPU/GPU**	**Deferred**	**Reference**
rspectra_sparse_arpack	arpack	sparse	RSpectra	R	CPU	no	(Lehoucq et al., 1998)
rapids_dense_exact	exact	dense	Rapids	Python	GPU	no	(Kalman, 1996)
scanpy_sparse_arpack	arpack	sparse	Scanpy	Python	CPU	no	(Lehoucq et al., 1998)
rapids_dense_jacobi	jacobi	dense	Rapids	Python	GPU	no	(Drmač and Veselić, 2008)
sciktlearn_sparse_IPCA	IPCA	sparse	SciktLearn	Python	CPU	no	(Ross et al., 2008)
scanpy_dense_arpack	arpack	dense	scanpy	Python	CPU	no	(Lehoucq et al., 1998)
bioc_sparse_def_random	random	sparse	BiocSingular	R	CPU	yes	(Halko et al., 2011)
bioc_sparsearray_def_random	random	sparse	BiocSingular	R	CPU	yes	(Halko et al., 2011)
scanpy_dense_random	random	dense	Scanpy	Python	CPU	no	(Halko et al., 2011)
scanpy_sparse_random	random	sparse	Scanpy	Python	CPU	no	(Halko et al., 2011)
sciktlearn_dense_IPCA	IPCA	dense	SciktLearn	Python	CPU	no	(Ross et al., 2008)
rspectra_dense_arpack	arpack	dense	RSpectra	R	CPU	no	(Lehoucq et al., 1998)
bioc_sparse_def_irlba	irlba	sparse	BiocSingular	R	CPU	yes	(Baglama and Reichel, 2005)
sciktlearn_dense_exact	exact	dense	SciktLearn	Python	CPU	no	(Kalman, 1996)
bioc_dense_random	random	dense	BiocSingular	R	CPU	no	(Halko et al., 2011)
bioc_sparse_random	random	dense	biocsingular	R	CPU	no	(Halko et al., 2011)
bioc_sparsearray_random	random	sparse	BioCsingular	R	CPU	no	(Halko et al., 2011)
bioc_hdf5_dense_random	random	hdf5	BioCsingular	R	CPU	no	(Halko et al., 2011)
bioc_sparse_irlba	irlba	sparse	biocsingular	R	CPU	no	(Baglama and Reichel, 2005)
bioc_sparse_def_exact	exact	sparse	biocsingular	R	CPU	yes	(Kalman, 1996)
bioc_dense_irlba	irlba	dense	BiocSingular	R	CPU	no	(Baglama and Reichel, 2005)
bioc_sparse_exact	exact	sparse	BiocSingular	R	CPU	no	(Kalman, 1996)
bioc_hdf5_dense_exact	exact	dense	BiocSingular	R	CPU	no	(Kalman, 1996)
bioc_hdf5_dense_irlba	irlba	hdf5	BiocSingular	R	CPU	no	(Baglama and Reichel, 2005)
bioc_dense_exact	exact	dense	BiocSingular	R	CPU	no	(Kalman, 1996)
bioc_sparsearray_exact	exact	sparse	BiocSingular	R	CPU	no	(Kalman, 1996)
rspectra_hdf5_dense_arpack	arpack	dense	RSpectra	R	CPU	no	(Lehoucq et al., 1998)
rspectra_hdf5_sparse_arpack	arpack	sparse	RSpectra	R	CPU	no	(Lehoucq et al., 1998)

Table S2: Comparison of various singular value decomposition (SVD) methods applied to dense or sparse data, specifying the library, programming language, computation type (CPU/GPU), use of deferred computation, and the ranking estimated using the Plackett–Luce model. Method names follow a naming convention that encodes the library, data type, and specific SVD algorithm used.

Methods	n cells	Algorithms	mean_time	sd_time	Matrix type	core_eng	software
bioc_hdf5_dense	100k	random	108.35	12.91	dense matrix	CPU	R
bioc_hdf5_dense	100k	exact	333.85	9.01	dense matrix	CPU	R
bioc_hdf5_dense	100k	irlba	676.50	32.81	dense matrix	CPU	R
rapids_dense	100k	exact	3.00	0.00	dense matrix	GPU	python
rapids_dense	100k	jacobi	3.00	0.00	dense matrix	GPU	python
scanpy_sparse	100k	random	6.20	0.42	sparse matrix	CPU	python
scanpy_sparse	100k	arpack	8.10	0.32	sparse matrix	CPU	python
sciktlearn_sparse	100k	IPCA	23.90	1.10	sparse matrix	CPU	python
rspectra_dense	100k	arpack	33.81	2.64	dense matrix	CPU	R
rspectra_sparse	100k	arpack	4.76	0.05	sparse matrix	CPU	R
sciktlearn_dense	100k	exact	35.80	2.35	dense matrix	CPU	python
bioc_dense	100k	random	62.59	0.41	dense matrix	CPU	R
bioc_dense	100k	exact	320.42	5.15	dense matrix	CPU	R
bioc_dense	100k	irlba	23.47	0.11	dense matrix	CPU	R
bioc_sparse_def	100k	random	8.65	0.03	sparse matrix	CPU	R
bioc_sparse_def	100k	exact	315.40	3.18	sparse matrix	CPU	R
bioc_sparse_def	100k	irlba	11.59	0.08	sparse matrix	CPU	R
bioc_sparse	100k	random	69.36	0.57	sparse matrix	CPU	R
bioc_sparse	100k	exact	319.23	5.57	sparse matrix	CPU	R
bioc_sparse	100k	irlba	11.72	0.08	sparse matrix	CPU	R
scanpy_dense	100k	random	7.10	0.57	dense matrix	CPU	python
scanpy_dense	100k	arpack	14.90	2.42	dense matrix	CPU	python
sciktlearn_dense	100k	IPCA	37.60	1.51	dense matrix	CPU	python
bioc_hdf5_sparse	100k	random	92.71	4.27	sparse matrix	CPU	R
bioc_hdf5_sparse	100k	exact	80.33	2.37	sparse matrix	CPU	R
bioc_hdf5_sparse	100k	irlba	1346.71	68.95	sparse matrix	CPU	R
rspectra_hdf5_dense	100k	arpack	1259.35	0.52	dense matrix	CPU	R
rspectra_hdf5_sparse	100k	arpack	2000.26	0.00	sparse matrix	CPU	R

Table S3: Computational Time for PCA benchmark for all methods presented in the work. (100k)

**Methods**	**n cells**	**algorithm**	**mean_time**	**sd_time**	**matrix type**	**core_eng**	**software**
bioc_hdf5_dense	500k	random	548.66	12.59	dense matrix	CPU	R
bioc_hdf5_dense	500k	exact	1687.71	72.66	dense matrix	CPU	R
bioc_hdf5_dense	500k	irlba	5506.23	214.87	dense matrix	CPU	R
rapids_dense	500k	exact	4.10	0.32	dense matrix	GPU	python
rapids_dense	500k	jacobi	4.00	0.00	dense matrix	GPU	python
scanpy_sparse	500k	random	33.70	1.95	sparse matrix	CPU	python
scanpy_sparse	500k	arpack	40.20	0.42	sparse matrix	CPU	python
sciktlearn_sparse	500k	IPCA	117.00	5.73	sparse matrix	CPU	python
rspectra_dense	500k	arpack	179.49	15.27	dense matrix	CPU	R
rspectra_sparse	500k	arpack	23.63	0.20	sparse matrix	CPU	R
sciktlearn_dense	500k	exact	192.00	8.07	dense matrix	CPU	python
bioc_dense	500k	random	309.98	1.37	dense matrix	CPU	R
bioc_dense	500k	exact	1664.83	9.19	dense matrix	CPU	R
bioc_dense	500k	irlba	126.88	0.60	dense matrix	CPU	R
bioc_sparse_def	500k	random	45.13	0.21	sparse matrix	CPU	R
bioc_sparse_def	500k	exact	1596.32	8.62	sparse matrix	CPU	R
bioc_sparse_def	500k	irlba	51.33	0.34	sparse matrix	CPU	R
bioc_sparse	500k	random	395.89	3.38	sparse matrix	CPU	R
bioc_sparse	500k	exact	1654.41	15.08	sparse matrix	CPU	R
bioc_sparse	500k	irlba	52.10	0.39	sparse matrix	CPU	R
scanpy_dense	500k	random	39.30	1.83	dense matrix	CPU	python
scanpy_dense	500k	arpack	85.80	11.79	dense matrix	CPU	python
sciktlearn_dense	500k	IPCA	197.80	1.99	dense matrix	CPU	python
bioc_hdf5_sparse	500k	random	381.18	17.72	sparse matrix	CPU	R
bioc_hdf5_sparse	500k	exact	400.42	12.33	sparse matrix	CPU	R
bioc_hdf5_sparse	500k	irlba	7142.68	182.73	sparse matrix	CPU	R
rspectra_hdf5_dense	500k	arpack	6213.26	7.51	dense matrix	CPU	R
rspectra_hdf5_sparse	500k	arpack	10754.67	0.00	sparse matrix	CPU	R

Table S4: Computational Time for PCA benchmark for all methods presented in the work. (500k)

Methods	n cells	Algorithm	mean_time	sd_time	MAtrix type	core_eng	software
bioc_hdf5_dense	1M	random	1147.51	30.52	dense matrix	CPU	R
bioc_hdf5_dense	1M	exact	3440.95	174.00	dense matrix	CPU	R
bioc_hdf5_dense	1M	irlba	12680.44	335.62	dense matrix	CPU	R
rapids_dense	1M	exact	5.00	0.00	dense matrix	GPU	python
rapids_dense	1M	jacobi	5.20	0.42	dense matrix	GPU	python
scanpy_sparse	1M	random	69.00	3.74	sparse matrix	CPU	python
scanpy_sparse	1M	arpack	82.00	0.47	sparse matrix	CPU	python
sciktlearn_sparse	1M	IPCA	222.30	7.01	sparse matrix	CPU	python
rspectra_dense	1M	arpack	376.11	31.68	dense matrix	CPU	R
rspectra_sparse	1M	arpack	47.41	0.46	sparse matrix	CPU	R
sciktlearn_dense	1M	exact	411.50	69.64	dense matrix	CPU	python
bioc_dense	1M	random	631.87	1.97	dense matrix	CPU	R
bioc_dense	1M	exact	3356.71	5.58	dense matrix	CPU	R
bioc_dense	1M	irlba	271.36	1.51	dense matrix	CPU	R
bioc_sparse_def	1M	random	94.47	0.35	sparse matrix	CPU	R
bioc_sparse_def	1M	exact	3226.88	22.44	sparse matrix	CPU	R
bioc_sparse_def	1M	irlba	163.69	0.95	sparse matrix	CPU	R
bioc_sparse	1M	random	925.95	5.16	sparse matrix	CPU	R
bioc_sparse	1M	exact	3343.25	22.82	sparse matrix	CPU	R
bioc_sparse	1M	irlba	163.28	0.31	sparse matrix	CPU	R
scanpy_dense	1M	random	81.70	2.87	dense matrix	CPU	python
scanpy_dense	1M	arpack	205.80	13.26	dense matrix	CPU	python
sciktlearn_dense	1M	IPCA	369.10	16.38	dense matrix	CPU	python
bioc_hdf5_sparse	1M	random	771.28	25.33	sparse matrix	CPU	R
bioc_hdf5_sparse	1M	exact	785.08	33.18	sparse matrix	CPU	R
bioc_hdf5_sparse	1M	irlba	17663.29	961.17	sparse matrix	CPU	R
rspectra_hdf5_dense	1M	arpack	13034.39	126.77	dense matrix	CPU	R
rspectra_hdf5_sparse	1M	arpack	22256.76	0.00	sparse matrix	CPU	R

Table S5: Computational Time for PCA benchmark for all methods presented in the work. (1M)

Methods	n cells	Algorithm	mean_time	sd_time	Matrix type	core_eng	software
bioc_hdf5_dense	1.3M	random	1515.55	47.78	dense matrix	CPU	R
bioc_hdf5_dense	1.3M	exact	4513.47	250.32	dense matrix	CPU	R
bioc_hdf5_dense	1.3M	irlba	17995.83	436.47	dense matrix	CPU	R
rapids_dense	1.3M	exact	7.50	0.53	dense matrix	GPU	python
rapids_dense	1.3M	jacobi	8.00	0.00	dense matrix	GPU	python
scanpy_sparse	1.3M	random	93.40	4.70	sparse matrix	CPU	python
scanpy_sparse	1.3M	arpack	106.60	0.97	sparse matrix	CPU	python
sciktlearn_sparse	1.3M	IPCA	292.40	9.88	sparse matrix	CPU	python
rspectra_dense	1.3M	arpack	467.73	25.02	dense matrix	CPU	R
rspectra_sparse	1.3M	arpack	61.96	0.92	sparse matrix	CPU	R
sciktlearn_dense	1.3M	exact	546.20	69.81	dense matrix	CPU	python
bioc_dense	1.3M	random	855.92	2.42	dense matrix	CPU	R
bioc_dense	1.3M	exact	4423.35	6.07	dense matrix	CPU	R
bioc_dense	1.3M	irlba	372.98	1.05	dense matrix	CPU	R
bioc_sparse_def	1.3M	random	122.01	0.63	sparse matrix	CPU	R
bioc_sparse_def	1.3M	exact	4281.73	22.22	sparse matrix	CPU	R
bioc_sparse_def	1.3M	irlba	173.29	1.22	sparse matrix	CPU	R
bioc_sparse	1.3M	random	1293.50	6.83	sparse matrix	CPU	R
bioc_sparse	1.3M	exact	4379.43	22.55	sparse matrix	CPU	R
bioc_sparse	1.3M	irlba	174.36	0.39	sparse matrix	CPU	R
scanpy_dense	1.3M	random	109.40	4.86	dense matrix	CPU	python
scanpy_dense	1.3M	arpack	263.70	16.61	dense matrix	CPU	python
sciktlearn_dense	1.3M	IPCA	520.80	18.94	dense matrix	CPU	python
bioc_hdf5_sparse	1.3M	random	811.56	11.06	sparse matrix	CPU	R
bioc_hdf5_sparse	1.3M	exact	763.67	18.25	sparse matrix	CPU	R
bioc_hdf5_sparse	1.3M	irlba	18659.81	1360.73	sparse matrix	CPU	R
rspectra_hdf5_dense	1.3M	arpack	17218.16	176.35	dense matrix	CPU	R
rspectra_hdf5_sparse	1.3M	arpack	22308.97	0.00	sparse matrix	CPU	R

Table S6: Computational Time for PCA benchmark for all methods presented in the work. (1.3M)

n cells	Methods	algorithm	mean_max_mem	sd_max_mem	matrix type	core_eng	soft
100k	bioc_hdf5	random	1329.894025	0.01	dense matrix	CPU	R
100k	bioc_hdf5	exact	4341.543908	11.6	dense matrix	CPU	R
100k	bioc_hdf5	irlba	49355.08551	0.01	dense matrix	CPU	R
100k	bioc_hdf5	random	1020.8476	0.01	sparse matrix	CPU	R
100k	bioc_hdf5	exact	5049.938	0.01	sparse matrix	CPU	R
100k	bioc_hdf5	irlba	26891.1733	0.01	sparse matrix	CPU	R
100k	bioc_dense	random	16210.72193	0	dense matrix	CPU	R
100k	bioc_dense	exact	22858.62363	0	dense matrix	CPU	R
100k	bioc_dense	irlba	49560.05276	18.88	dense matrix	CPU	R
100k	bioc_sparse	random	9037.863437	0	sparse matrix	CPU	R
100k	bioc_sparse	exact	14623.11574	0	sparse matrix	CPU	R
100k	bioc_sparse	irlba	37400.22575	18.88	sparse matrix	CPU	R
100k	bioc_sparse_def	random	6310.129139	0	sparse matrix	CPU	R
100k	bioc_sparse_def	exact	14843.84114	0	sparse matrix	CPU	R
100k	bioc_sparse_def	irlba	38855.68576	12181.2	sparse matrix	CPU	R
100k	scanpy_dense	random	1722.33429	0.3140134532	dense matrix	CPU	python
100k	scanpy_dense	arpack	1652.267075	0.6657700317	dense matrix	CPU	python
100k	sciktlearn_dense	IPCA	1760.367203	0.5872789718	dense matrix	CPU	python
100k	rapids_dense	exact	4920	329.3090409	dense matrix	GPU	python
100k	rapids_dense	jacobi	5120	413.1182236	dense matrix	GPU	python
100k	scanpy_sparse	random	1750.650406	2.317954596	sparse matrix	CPU	python
100k	scanpy_sparse	arpack	986.8175507	0.2107660095	sparse matrix	CPU	python
100k	sciktlearn_sparse	IPCA	1116.742706	31.31881767	sparse matrix	CPU	python
100k	rspectra_dense	arpack	12133.5152	0	dense matrix	CPU	R
100k	rspectra_sparse	arpack	5263.775693	0	sparse matrix	CPU	R
100k	sciktlearn_dense	exact	2251.295471	405.3892638	dense matrix	CPU	python

Table S7: Memory usage for PCA benchmark for all methods presented in the work. (100k)

n cells	Methods	algorithm	mean_max_mem	sd_max_mem	matrix type	core_eng	soft
500k	bioc_hdf5	random	2879.39	0.01	dense matrix	CPU	R
500k	bioc_hdf5	exact	16523.93	0.01	dense matrix	CPU	R
500k	bioc_hdf5	irlba	49259.32	27.27	dense matrix	CPU	R
500k	bioc_hdf5	random	2530.24	0.01	sparse matrix	CPU	R
500k	bioc_hdf5	exact	12821.22	0.01	sparse matrix	CPU	R
500k	bioc_hdf5	irlba	7073.86	0.01	sparse matrix	CPU	R
500k	bioc_dense	random	19384.29	0.00	dense matrix	CPU	R
500k	bioc_dense	exact	27037.59	0.00	dense matrix	CPU	R
500k	bioc_dense	irlba	49581.63	0.00	dense matrix	CPU	R
500k	bioc_sparse	random	9632.38	0.00	sparse matrix	CPU	R
500k	bioc_sparse	exact	20630.80	0.00	sparse matrix	CPU	R
500k	bioc_sparse	irlba	38742.98	0.00	sparse matrix	CPU	R
500k	bioc_sparse_def	random	9050.79	0.00	sparse matrix	CPU	R
500k	bioc_sparse_def	exact	20625.30	0.00	sparse matrix	CPU	R
500k	bioc_sparse_def	irlba	40071.07	12567.15	sparse matrix	CPU	R
500k	scanpy_dense	random	7735.24	4.25	dense matrix	CPU	python
500k	scanpy_dense	arpack	7375.11	0.29	dense matrix	CPU	python
500k	sciktlearn_dense	IPCA	7723.03	0.69	dense matrix	CPU	python
500k	rapids_dense	exact	7720.00	3661.45	dense matrix	GPU	python
500k	rapids_dense	jacobi	7000.00	573.49	dense matrix	GPU	python
500k	scanpy_sparse	random	7925.61	0.38	sparse matrix	CPU	python
500k	scanpy_sparse	arpack	4073.94	0.29	sparse matrix	CPU	python
500k	sciktlearn_sparse	IPCA	4215.45	215.38	sparse matrix	CPU	python
500k	rspectra_dense	arpack	15385.56	0.00	dense matrix	CPU	R
500k	rspectra_sparse	arpack	5467.15	0.00	sparse matrix	CPU	R
500k	sciktlearn_dense	exact	10598.58	2289.88	dense matrix	CPU	python

Table S8: Memory usage for PCA benchmark for all methods presented in the work. (500k)

n cells	Methods	Algorithm	Mean Max Mem	SD Max Mem	Matrix Type	Core Eng	Soft
1M	bioc_hdf5	random	4953.075776	0.01	dense matrix	CPU	R
1M	bioc_hdf5	exact	32601.15021	0.01	dense matrix	CPU	R
1M	bioc_hdf5	irlba	37319.30883	240.21	dense matrix	CPU	R
1M	bioc_hdf5	random	4634.66163	0.01	sparse matrix	CPU	R
1M	bioc_hdf5	exact	25186.17480	0.01	sparse matrix	CPU	R
1M	bioc_hdf5	irlba	3234,25666	0.01	sparse matrix	CPU	R
1M	bioc_dense	random	19458.31991	0	dense matrix	CPU	R
1M	bioc_dense	exact	38645.91365	0	dense matrix	CPU	R
1M	bioc_dense	irlba	42516.13618	0.25	dense matrix	CPU	R
1M	bioc_sparse	random	12854.02404	0	sparse matrix	CPU	R
1M	bioc_sparse	exact	32239.12414	0	sparse matrix	CPU	R
1M	bioc_sparse	irlba	41376.78263	0.01	sparse matrix	CPU	R
1M	bioc_sparse_def	random	10873.2858	0	sparse matrix	CPU	R
1M	bioc_sparse_def	exact	29864.4428	0	sparse matrix	CPU	R
1M	bioc_sparse_def	irlba	41082.69968	12884.5	sparse matrix	CPU	R
1M	scanpy_dense	random	15202.94571	0.4429	dense matrix	CPU	python
1M	scanpy_dense	arpack	14530.27153	0.5370	dense matrix	CPU	python
1M	sciktlearn_dense	IPCA	15061.72638	0.6498	dense matrix	CPU	python
1M	rapids_dense	exact	10720	413.1182	dense matrix	GPU	python
1M	rapids_dense	jacobi	11840	1413.5849	dense matrix	GPU	python
1M	scanpy_sparse	random	15576.70822	0.4238	sparse matrix	CPU	python
1M	scanpy_sparse	arpack	7930.33981	0.1863	sparse matrix	CPU	python
1M	sciktlearn_sparse	IPCA	7914.76173	0.3344	sparse matrix	CPU	python
1M	rspectra_dense	arpack	19555.02417	0	dense matrix	CPU	R
1M	rspectra_sparse	arpack	5872.58629	0	sparse matrix	CPU	R
1M	sciktlearn_dense	exact	21041.25252	4612.26238	dense matrix	CPU	python

Table S9: Memory usage for PCA benchmark for all methods presented in the work. (1M)

n cells	Methods	algorithm	mean_max_mem	sd_max_mem	matrix type	core_eng	soft
1.3M	bioc_hdf5	random	7041.36567	0.01	dense matrix	CPU	R
1.3M	bioc_hdf5	exact	40995.85332	0.01	dense matrix	CPU	R
1.3M	bioc_hdf5	irlba	38906.17193	600.52	dense matrix	CPU	R
1.3M	bioc_hdf5	random	5430.44560	0.01	sparse matrix	CPU	R
1.3M	bioc_hdf5	exact	26411.33458	0.01	sparse matrix	CPU	R
1.3M	bioc_hdf5	irlba	4115.74381	0.01	sparse matrix	CPU	R
1.3M	bioc_dense	random	23396.41941	0	dense matrix	CPU	R
1.3M	bioc_dense	exact	48543.04005	0	dense matrix	CPU	R
1.3M	bioc_dense	irlba	44921.05819	0	dense matrix	CPU	R
1.3M	bioc_sparse	random	13994.26933	0	sparse matrix	CPU	R
1.3M	bioc_sparse	exact	40612.50722	0	sparse matrix	CPU	R
1.3M	bioc_sparse	irlba	44815.26027	0.25	sparse matrix	CPU	R
1.3M	bioc_sparse_def	random	15338.3199	0	sparse matrix	CPU	R
1.3M	bioc_sparse_def	exact	42124.57184	0	sparse matrix	CPU	R
1.3M	bioc_sparse_def	irlba	24266.42509	7610.52	sparse matrix	CPU	R
1.3M	scanpy_dense	random	19767.25197	0.4525564595	dense matrix	CPU	python
1.3M	scanpy_dense	arpack	19419.6537	448.6105733	dense matrix	CPU	python
1.3M	sciktlearn_dense	IPCA	19640.01923	0.56599918	dense matrix	CPU	python
1.3M	rapids_dense	exact	14640	539.9588462	dense matrix	GPU	python
1.3M	rapids_dense	jacobi	15480	1208.120671	dense matrix	GPU	python
1.3M	scanpy_sparse	random	20260.21881	0.2514549124	sparse matrix	CPU	python
1.3M	scanpy_sparse	arpack	10275.97389	0.2320058763	sparse matrix	CPU	python
1.3M	sciktlearn_sparse	IPCA	10626.25923	113.9357804	sparse matrix	CPU	python
1.3M	rspectra_dense	arpack	22396.61597	0	dense matrix	CPU	R
1.3M	rspectra_sparse	arpack	6123.724961	0	sparse matrix	CPU	R
1.3M	sciktlearn_dense	exact	29338.12294	12.39934045	dense matrix	CPU	python

Table S10: Memory usage for PCA benchmark for all methods presented in the work. (1.3M)

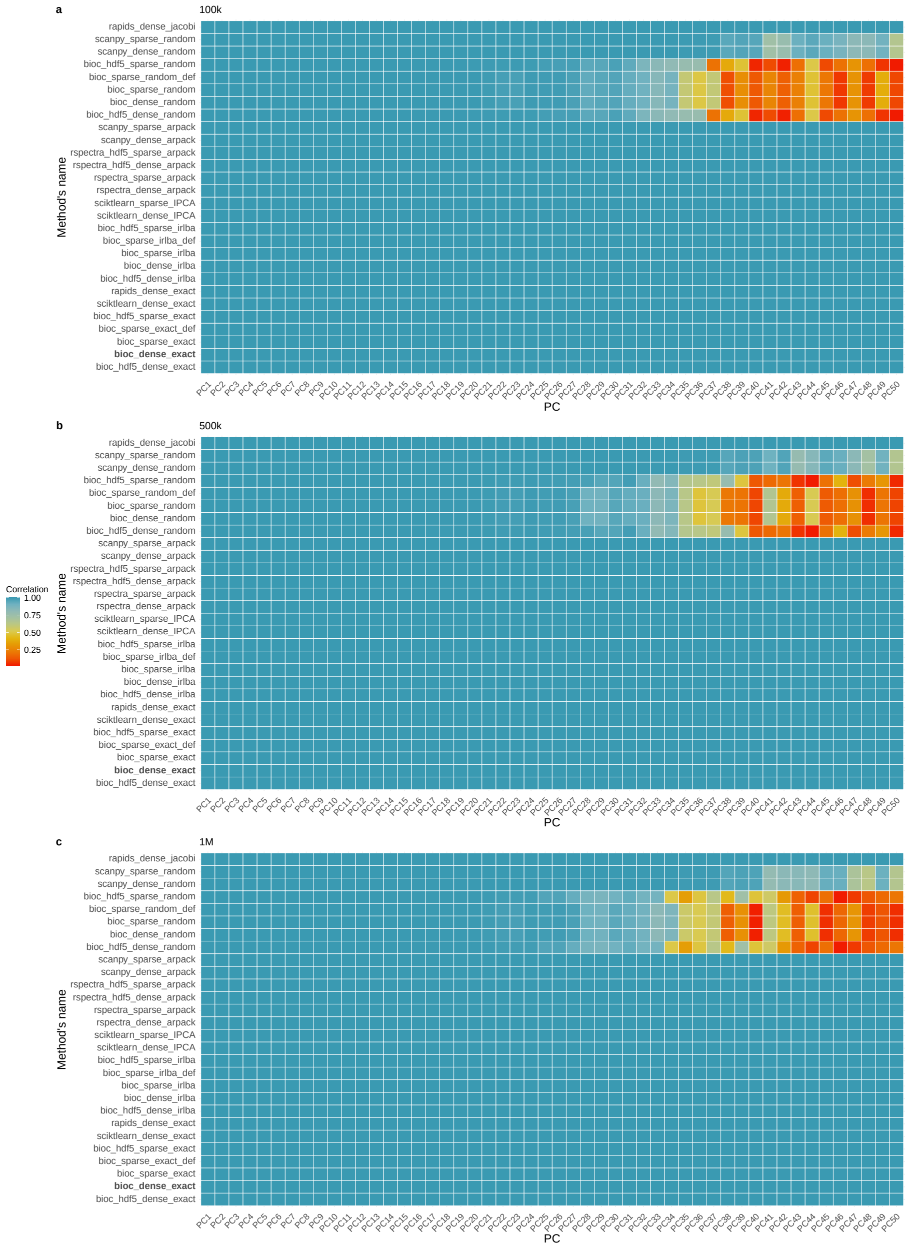

Figure S2: **Correlation heatmaps between principal components (PC)** Correlation heatmaps between principal components (PCs) obtained using different PCA algorithms and the Exact Algorithm as reference. Each row represents a different combination of PCA algorithm and input matrix, and each column a principal component (PC1–PC50). The color indicates the Pearson correlation between the PC obtained from each method and the corresponding PC from the exact reference. Panels correspond to datasets containing (a) 100k, (b) 500k, and (c) 1M cells, respectively.

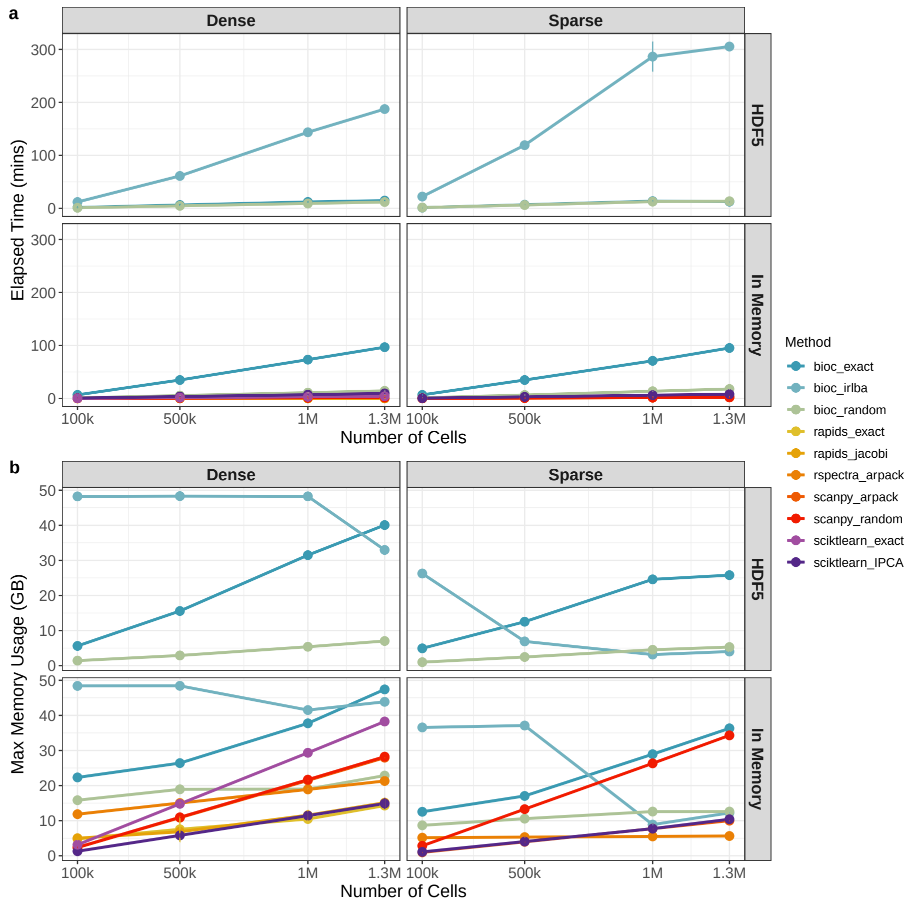

Figure S3: **Scalability Assessment of PCA Methods by Input Dimensions, Runtime, and Memory Consumption** (a) Elapsed time (in minutes) required to perform principal component analysis (PCA) across a range of dataset sizes (100k, 500k, 1M, 1.3M cells), using different combinations of methods, matrix formats (dense or sparse), and storage types (in-memory or HDF5). (b) Maximum memory usage (in GB) required to perform principal component analysis (PCA) across a range of dataset sizes (100k, 500k, 1M, 1.3M cells), using different combinations of methods, matrix formats (dense or sparse), and storage types (in-memory or HDF5).

	PC1	PC2	PC3	PC4	PC5	PC6	PC7	PC8	PC9	PC10
100k	75.28	17.13	1.71	1.15	1.04	0.87	0.51	0.30	0.29	0.22
500k	74.36	17.75	1.78	1.21	1.04	0.92	0.53	0.32	0.29	0.23
1M	74.47	17.72	1.77	1.20	1.03	0.92	0.53	0.32	0.28	0.23
1.3M	74.40	17.76	1.78	1.21	1.03	0.92	0.53	0.32	0.29	0.23

Table S11: Explained variance percentages for the bioc_dense_random method. Each value represents the proportion of total variance captured by the corresponding singular value component, computed using a randomized SVD algorithm on dense matrices within the BiocSingular framework. (PC1 – PC10)

	PC11	PC12	PC13	PC14	PC15	PC16	PC17	PC18	PC19	PC20
100k	0.19	0.14	0.13	0.08	0.07	0.07	0.06	0.05	0.05	0.04
500k	0.20	0.15	0.13	0.08	0.07	0.07	0.06	0.06	0.05	0.05
1M	0.20	0.15	0.13	0.09	0.08	0.07	0.06	0.06	0.05	0.05
1.3M	0.20	0.15	0.13	0.09	0.08	0.07	0.06	0.06	0.05	0.05

Table S12: Explained variance percentages for the bioc_dense_random method. Each value represents the proportion of total variance captured by the corresponding singular value component, computed using a randomized SVD algorithm on dense matrices within the BiocSingular framework. (PC11 – PC20)

	PC21	PC22	PC23	PC24	PC25	PC26	PC27	PC28	PC29	PC30
100k	0.04	0.04	0.03	0.03	0.03	0.03	0.03	0.03	0.02	0.02
500k	0.05	0.04	0.04	0.04	0.04	0.03	0.03	0.03	0.03	0.03
1M	0.04	0.04	0.04	0.03	0.03	0.03	0.03	0.03	0.03	0.03
1.3M	0.04	0.04	0.04	0.04	0.03	0.03	0.03	0.03	0.03	0.03

Table S13: Explained variance percentages for the bioc_dense_random method. Each value represents the proportion of total variance captured by the corresponding singular value component, computed using a randomized SVD algorithm on dense matrices within the BiocSingular framework. (PC21 – PC30)

	PC31	PC32	PC33	PC34	PC35	PC36	PC37	PC38	PC39	PC40
100k	0.02	0.02	0.02	0.02	0.02	0.02	0.02	0.01	0.01	0.01
500k	0.02	0.02	0.02	0.02	0.02	0.02	0.02	0.02	0.02	0.01
1M	0.02	0.02	0.02	0.02	0.02	0.02	0.02	0.02	0.02	0.01
1.3M	0.02	0.02	0.02	0.02	0.02	0.02	0.02	0.02	0.02	0.01

Table S14: Explained variance percentages for the bioc_dense_random method. Each value represents the proportion of total variance captured by the corresponding singular value component, computed using a randomized SVD algorithm on dense matrices within the BiocSingular framework. (PC31 – PC40)

	PC41	PC42	PC43	PC44	PC45	PC46	PC47	PC48	PC49	PC50
100k	0.01	0.01	0.01	0.01	0.01	0.01	0.01	0.01	0.01	0.01
500k	0.01	0.01	0.01	0.01	0.01	0.01	0.01	0.01	0.01	0.01
1M	0.01	0.01	0.01	0.01	0.01	0.01	0.01	0.01	0.01	0.01
1.3M	0.01	0.01	0.01	0.01	0.01	0.01	0.01	0.01	0.01	0.01

Table S15: Explained variance percentages for the bioc_dense_random method. Each value represents the proportion of total variance captured by the corresponding singular value component, computed using a randomized SVD algorithm on dense matrices within the BiocSingular framework. (PC41 – PC50)

Size	Methods	Unoptimized Elapsed Time Medium03 R	Optimized Elapsed Time Medium03 R	xen6 Unoptimized	xen6 Optimized
100k	bioc_exact_dense	156.14	16.255	408	11.876
500k	bioc_exact_dense	858.491	88.155	2088	76.858
1M	bioc_exact_dense	1655.495	165.451	4392	151.293
1.3M	bioc_exact_dense	2125.143	233.62	5916	197.526

Table S16: Computation times (in seconds) for exact SVD using different LAPACK/BLAS configurations in R.

	step	Seurat	OSCA	Scanpy	Rapids	scrapper
1.3M	find mitochondrial gene	15.544	0.47	98.8152	4.685	1010.838
	filter	139.8	637.84	209.1486	272.8392	
	normalization	62.4	2674.48	47.0995	60.6309	0.1137
	hgv	69	3038.39	55.6003	90.9464	1393.488
	scaling	67.44	0	15.2298	11.978	0
	PCA	7344	1350.96	85.1788	43.69	536.052
	t-sne	5472	0.00	4513.9915	101.72	13638.96
	umap	1180.8	1200.00	1766.2184	350.452	1581.12
	louvain	2657.4	648.326	572.534	388.746	1207.2
	leiden	2636.16	480.00	745.631	392.798	1139.4
cb	find mitochondrial gene	0.9603	8.9845	1.4657	0	0.8605
	filter	1.6499		1.7012	0.455	0
	normalization	0.9406	15.3187	1.4122	0.006	0.0613
	hgv	1.3899	0.6372	1.2622	0.488	1.1154
	scaling	9.4327		0.4977	0.043	0
	PCA	3.0979	9.4448	0.365	0.565	0.763
	t-sne	24.4497	48.0701	24.1626	0.952	17.6854
	umap	17.5386	15.6646	20.581	5.36	11.6143
	louvain	4.5575	14.8174	0.5714	2.052	3.5234
	leiden	4.7349	8.7005	0.4191	0.097	1.7955
sc_mixology	find mitochondrial gene	0.7783	9.211	0.8896	0	2.2526
	filter	1.4675		1.5183	1.087	0
	normalization	0.4666	3.9137	0.2358	0.006	0.0584
	hgv	0.6548	0.6936	0.1535	0.1178	0.596
	scaling	0.9108	0	0.2961	0.04	
	PCA	0.4304	3.9362	0.2058	0.075	0.3807
	t-sne	2.8912	22.3044	2.105	0.045	7.0366
	umap	4.5184	8.5114	9.1539	2.7556	5.2801
	louvain	0.7204	2.0218	0.0268	2.965	0.8662
	leiden	0.6141	1.1177	0.1284	0.089	0.6106
BE1	find mitochondrial gene	4.4057	11.5306	6.819	1.8119	0.6141
	filter	6.1036	0.049	19.8166	4.8015	0
	normalization	4.8742	7.5891	11.3122	1.1635	1.3935
	hgv	10.0936	0.8263	10.3254	0.375	1.263
	scaling	93.576	0	7.7532	1.865	
	PCA	7.7419	52.245	11.1524	2.5294	4.8703
	t-sne	128.982	182.4	102.7969	3.8564	61.326
	umap	46.1377	29.2787	67.1117	6.1052	30.6672
	louvain	24.0327	103.956	5.1166	1.0125	13.9878
	leiden	25.3245	29.7326	2.8551	0.7038	6.4761

Table S17: Computational time for each scRNA-seq workflow and each database in input

Dataset	Sample	Seurat	OSCA	Scrapper	Scanpy	rapids_singlecell
BE1	A549	0.9002	0.9996	0.9997	0.8206	0.8206
	CCL-185-IG	0.8941	0.9947	0.9947	0.8279	0.8279
	CRL5868	0.9925	0.9966	0.9971	0.9947	0.9947
	DV90	0.9972	0.9988	0.9984	0.9977	0.9977
	HCC78	0.9982	0.9799	0.9796	0.9986	0.9986
	HTB178	0.9970	0.9750	0.9839	0.9957	0.9957
	PBMCs	0.9943	0.9567	0.9631	0.9981	0.9981
	PC9	0.9966	0.9989	0.9993	0.9911	0.9911
cb	B-cells	1.0000	0.9821	0.9824	1.0000	1.0000
	CD4 T-cells	0.7577	0.5181	0.5285	0.6578	0.6578
	CD8 T-cells	0.7557	0.5418	0.5519	0.6372	0.6372
	Monocytes CD14+	0.9965	0.9545	0.9517	0.9434	0.9434
	Monocytes CD16+	0.9132	0.9424	0.9349	0.9802	0.9802
	Natural Killers	0.9107	0.9435	0.9392	0.9930	0.9930
	Precursors	1.0000	0.9497	0.9481	1.0000	1.0000
	T-cells	0.7487	0.6420	0.6162	0.6642	0.6642
sc_mix	A549	1.0000	0.9994	0.9995	1.0000	1.0000
	H1975	0.9535	0.9827	0.9824	0.9593	0.9593
	H2228	0.9987	0.9992	0.9992	0.9987	0.9987
	H838	0.9929	0.9976	0.9975	1.0000	1.0000
	HCC827	0.9900	0.9966	0.9962	0.9636	0.9636

Table S18: **Average cell type purity across datasets and workflows.**The table reports the mean neighbor purity values computed for each cell type across the BE1, cb, and sc_mix datasets, using five single-cell analysis workflows (Seurat, OSCA, Scrapper, Scanpy, and rapidssc_singlecell).

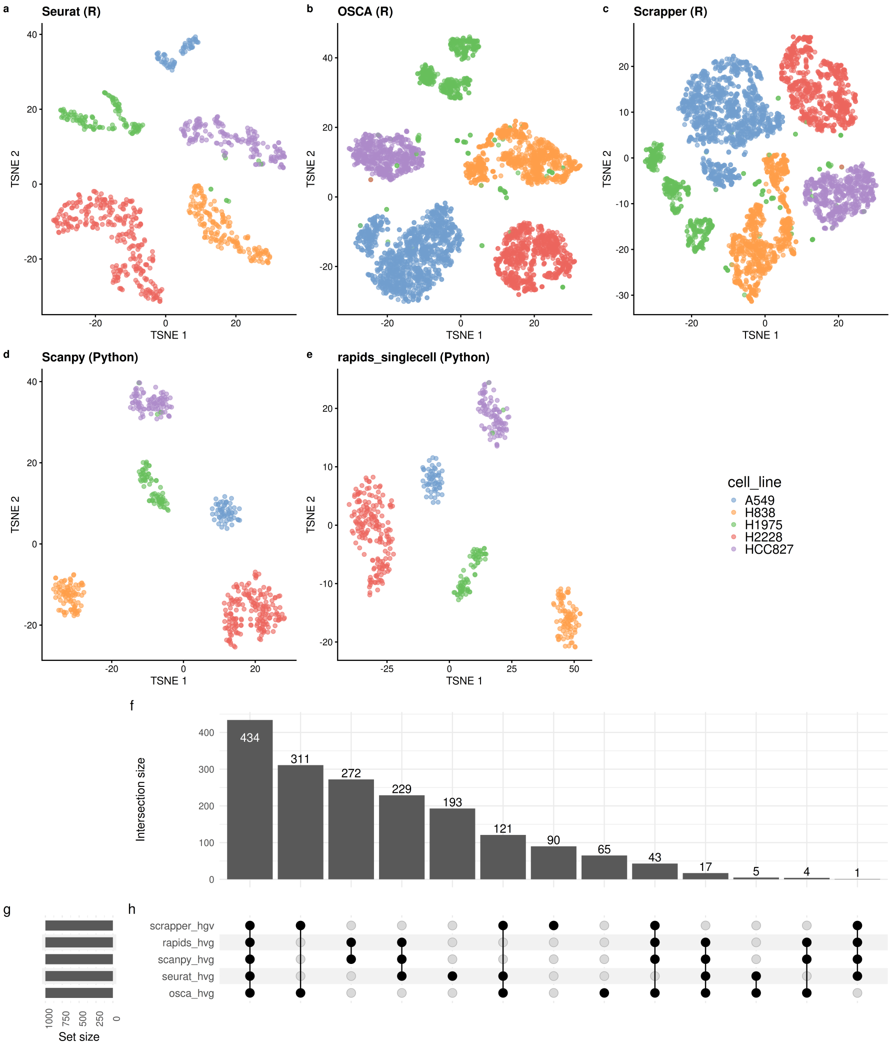

Figure S4: **Dimensionality reduction and feature selection comparison using the sc_mix dataset.** Dimensionality reduction and feature selection comparison using the sc_mix dataset. (a–e) t-SNE embeddings of the sc_mix dataset colored by sample identity, generated using five different single-cell workflows: Seurat (a), OSCA (b), Scrapper (c), Scanpy (d), and rapids_singlecell (e). Each workflow applies its own normalization and highly variable gene (HVG) selection procedure prior to dimensionality reduction. (f–h) UpSet plot showing the intersection of HVG sets selected by each workflow. Panel (f) indicates the size of each intersection set, (g) shows the number of genes selected per method (set size), and (h) depicts the overlap structure across methods.

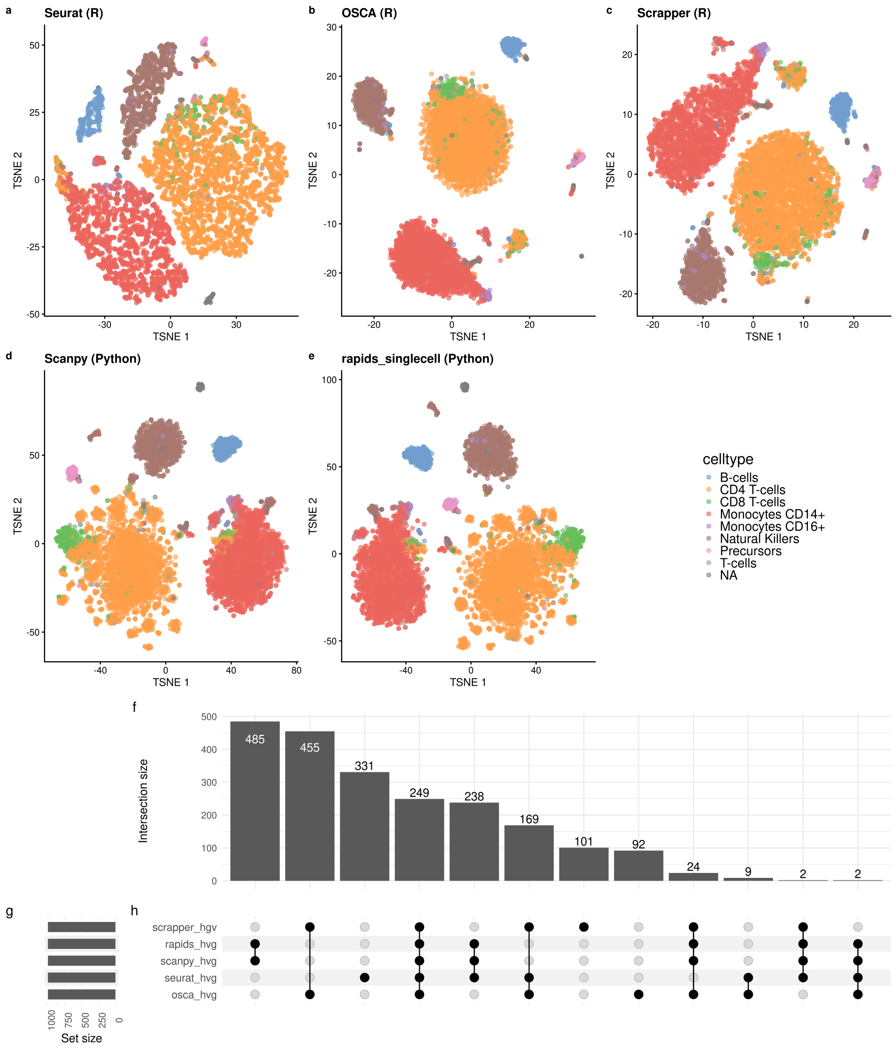

Figure S5: **Dimensionality reduction and feature selection comparison using the cb dataset.** (a–e) t-SNE embeddings of the cb dataset colored by sample identity, generated using five different single-cell workflows: Seurat (a), OSCA (b), Scrapper (c), Scanpy (d), and rapids_singlecell (e). Each workflow applies its own normalization and highly variable gene (HVG) selection procedure prior to dimensionality reduction. (f–h) UpSet plot showing the intersection of HVG sets selected by each workflow. Panel (f) indicates the size of each intersection set, (g) shows the number of genes selected per method (set size), and (h) depicts the overlap structure across methods.

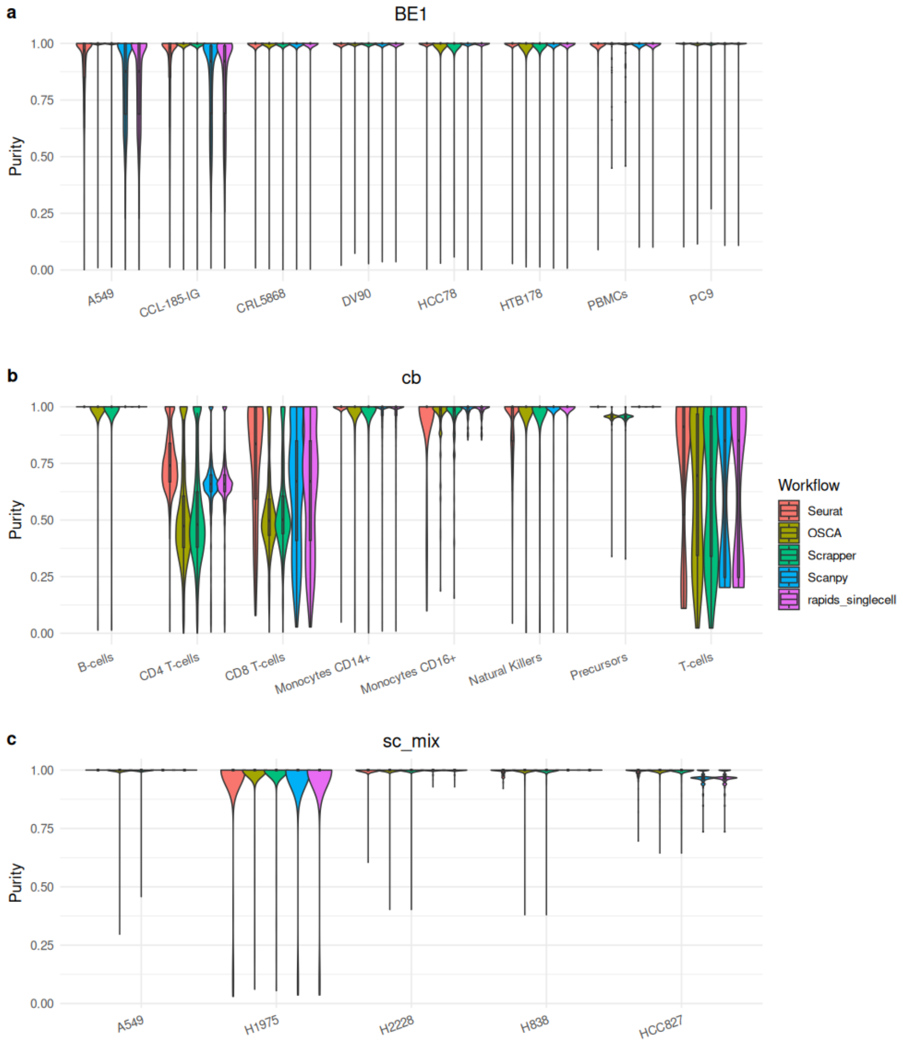

Figure S6: Comparison of clustering purity across single-cell analysis workflows on three datasets: (a) BE1, (b) cb, (c) sc_mix. Violin plots represent the distribution of clustering purity for each sample or cell type across five analysis workflows: Seurat, OSCA, Scrapper, Scanpy, and rapids_singlecell.

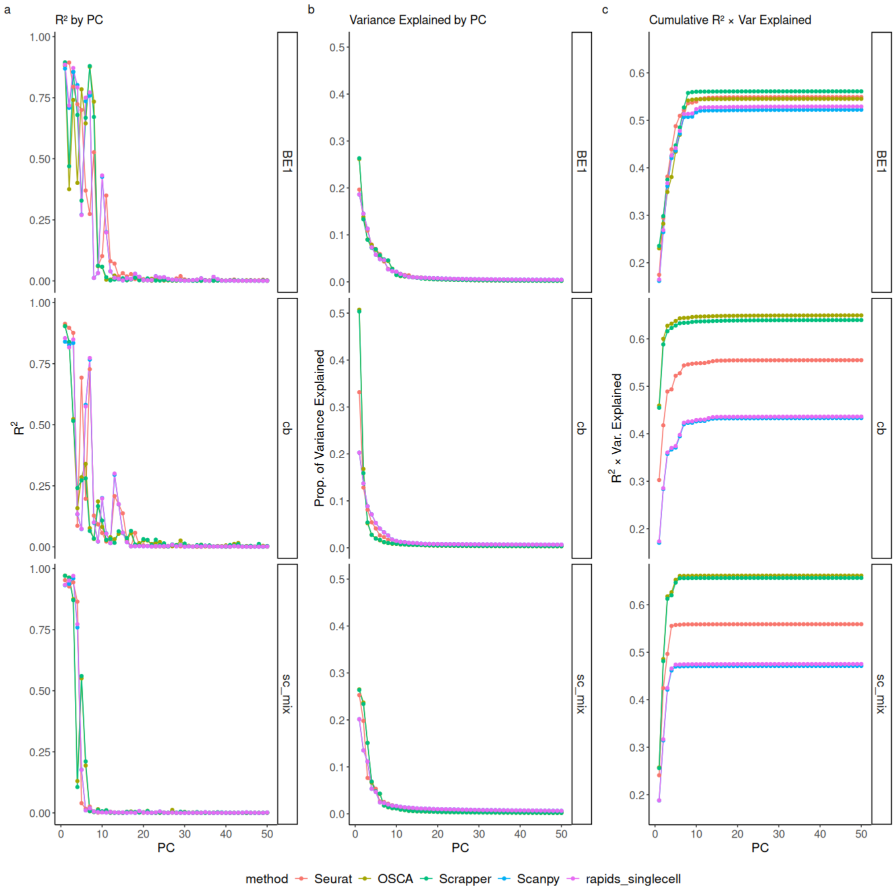

Figure S7: **Comparison of dimensionality reduction performance across single-cell workflows.** Line plots show three performance metrics computed across principal components (PCs) for three datasets: BE1, cb, and sc_mix (rows), and five workflows: Seurat, OSCA, Scrapper, Scanpy, and rapids_singlecell (colors). (a) R^2 by PC: the proportion of variance in the cell-type labels explained by each PC. (b) Variance Explained by PC: the proportion of total variance in the gene expression data captured by each PC. (c) Cumulative $R^2 \times$ Variance Explained: a composite metric reflecting both biological signal and data structure captured by the PCs.
